# Intramolecular Design of Poly(ethylene oxide) for Solid-State Electrolytes and Next-Generation High-Energy Batteries

**DOI:** 10.1007/s40820-026-02161-4

**Published:** 2026-05-18

**Authors:** Shijun Zhang, Ruiliu Jia, Xiaoyu Ji, Ziqing Zeng, Lei Li, Lijun Fu, Jianjun Zhang, Bin Chen, Yen Wei, Henghui Xu, Yang Yang, Guanglei Cui

**Affiliations:** 1 National Key Laboratory of Electromagnetic Energy, Naval University of Engineering, Wuhan, 430033 People’s Republic of China; 2 Qingdao Industrial Energy Storage Research Institute, Qingdao Institute of Bioenergy and Bioprocess Technology, Chinese Academy of Sciences, Qingdao, 266101 People’s Republic of China; 3 Institute of Nuclear and New Energy Technology, Tsinghua University, Beijing, 100084 People’s Republic of China; 4 Laboratory of Power and Energy Storage Batteries, School of Materials Science and Engineering, Huazhong University of Science and Technology, Wuhan, 430033 People’s Republic of China; 5 East Lake Laboratory, Wuhan, 430204 People’s Republic of China; 6 The Key Laboratory of Bioorganic Phosphorus Chemistry & Chemical Biology (Ministry of Education), Department of Chemistry, Tsinghua University, Beijing, 100084 People’s Republic of China

**Keywords:** Poly(ethylene oxide)(PEO), Molecular design, Solid-state electrolyte, High-energy batteries

## Abstract

Solid-state electrolytes (SSEs) are key materials for next-generation high-energy batteries because of their enhanced chemical and mechanical stabilities. Poly(ethylene oxide) (PEO)-based solid polymer electrolytes (SPEs) exhibit great physical contact with electrodes, electrochemical compatibility with lithium (Li) metal anodes, as well as easy processibility and high economic efficiency, having become the pioneer and one frontrunner for developing all-solid-state high-energy batteries. However, PEO-based SPEs also suffer from a trade-off between ionic conductivity and mechanical strength, an insufficient cationic transference number, and a weak high-voltage stability, limiting their practical achievement in desirable power and energy density. Herein, we present a comprehensive overview on the intramolecular design strategies of PEO, which has the potential to fundamentally tackle above challenges compared to the intermolecular plasticizer or ceramic blending approaches. Topological and chemical designs for target mechano-electro-chemical performance are classified and summarized in detail. On this basis, a perspective on the unconquered issues and future directions is proposed, providing guidance for the design and application of high-performance SSEs for next-generation high-energy batteries, with special emphasis on the rational integration of intramolecular and intermolecular methods and the development of advanced manufacture techniques for flexible yet robust thin films.

## Highlights

- This review summarizes the intramolecular design strategies of poly(ethylene oxide) (PEO)-based solid polymer electrolytes (SPEs) for next-generation high-energy batteries.
- Guided by bottleneck issues, this work categorized the intramolecular design into two approaches: topology and chemistry, with corresponding methodology, structure–property relationship, and underlying mechanism being clarified.
- Forward-looking perspective on current challenges and future research directions of PEO-based SPEs is carefully proposed.

## Graphical abstract

**Figure:**
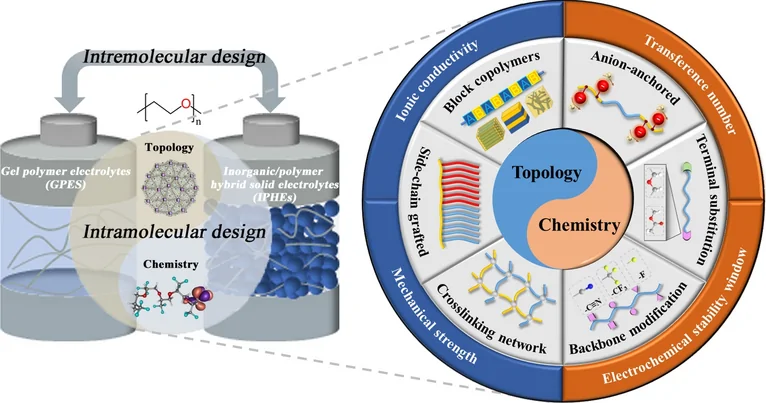

## Introduction

Unprecedented advance in electrified transportation, intelligent robots, and wearable electronics puts electrochemical energy storage devices much closer to human’s life, stimulating an urgent need for powerful yet safe batteries with higher energy density. Major governments have brought batteries with 400~500 Wh kg^−1^ into national development plans and aim to implement commercialization by 2030 [1]. One sought-after way to the ambitiousness is substituting graphite anodes of lithium-ion batteries (LIBs) with metallic Li anode, fabricating Li metal batteries (LMBs) that can achieve energy density beyond 500 Wh kg^−1^ [2–5]. As a trade-off, the challenge of next-generation high-energy batteries is the uncontrolled side reactions between highly active Li anodes and liquid electrolytes, causing anode powdering and electrolyte depletion, which significantly erode the cycling stability and safety [6–8]. In this context, SSEs are emerging as one ideal solution to these challenges, taking advantages of their enhanced chemical and mechanical stabilities [9–13].

Poly(ethylene oxide) (PEO), one of the earliest discovered alkali-metal ion conductors, exhibits high dielectric constant [14], favorable reductive stability to Li anode, good interfacial contact with electrodes, and easy processability, having been intensely studied with the development of solid-state batteries [15–17]. In 2011, French *Bolloré* corporation applied PEO doped with lithium bis(trifluoromethanesulfonyl)imide (LiTFSI) as SSEs, achieving the first application of solid-state LMBs in electric vehicles (EVs), *Bluecar* [18]. However, PEO delivers low room-temperature (RT) ionic conductivity (~ 10^−7^ S cm^−1^) and cationic transference number (*t*_+_, ~ 0.2), limiting cell’s available energy and power density [14, 19]. Temperatures over 60 °C are needed to soften the crystallization of PEO and increase ionic conductivity to 10^−4^ S cm^−1^ level, but a heating system inevitably induces the extra energy consumption and weight. While heating improves the ionic conductivity, moreover, the PEO matrix transforms from solid to viscoelastic state, resulting in a remarkable decline in mechanical stability, which is at odds with the need for suppressing dendrite growth and battery shorting. Besides, PEO exhibits a moderate oxidation potential (*E*_ox_) at ~ 3.8 V versus Li/Li^+^ [20], which matches well with lithium iron phosphate (LFP) cathode but fails to steadily work with state-of-the-art high-voltage cathodes, such as Li-rich manganese-based oxides [21–24] and nickel–cobalt–manganese oxides (NCM) [25–28]. As a result, the *Bluecar* battery using solid PEO-LiTFSI electrolyte, Li anode, and LFP cathode achieves energy density of merely 120 Wh kg^−1^ [29].

**Fig. 1:**
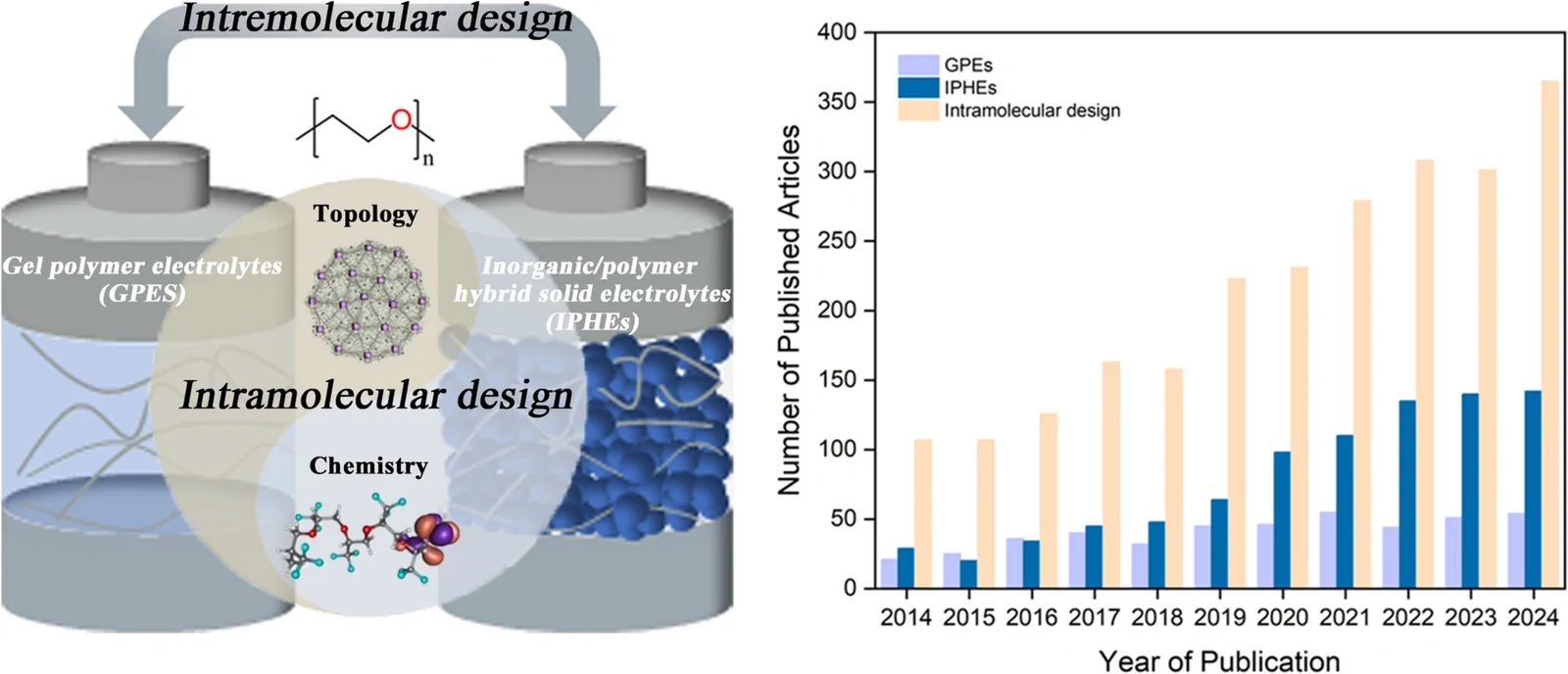
Characteristics and publication trends of inter-/intramolecular structure design in PEO-based SPEs. Statistical data for the presented publications are from the Web of Science Core Collection

Fundamentally, ion transport in SPEs, unlike solid inorganic electrolytes through lattice defects or grain boundaries [10, 30–32], relies heavily on the segmental relaxation of amorphous PEO chains with free volume [33, 34]. Such strong coupling indicates that an ionic conductivity as high as 10^−4^ S cm^−1^ requires a segmental mobility to be (0.25~2.5) × 10^9^ s^−1^ [35]. In sharp contrast, the relaxation rate of amorphous PEO segments is 10⁶~10⁷ s^−1^, which is 2~3 order of magnitudes slower than the required value. Moreover, most of PEO segments are confined in the crystalline structure at RT, which can hardly contribute to ion conduction. Therefore, lowering glass transition temperature (*T*_g_) and mitigating crystallization to facilitate the PEO segmental mobility are the most fundamental principle to achieve high-performance PEO-based SPEs, which can be classified into intermolecular and intramolecular categories (Fig. 1).

One common intermolecular strategy is adding plasticizers into the polymer matrix [36, 37]. The plasticizers include organic solvents of Les [38, 39], plastic crystals, such as succinonitrile (SN) [40–42]. Plasticizers can reduce the crystallinity and increase the free volume of polymer chains, improving ionic conductivity of PEO-based SPEs to 10^−4^~10^−3^ S cm^−1^ However, this method employs flammable solvents and sacrifices the mechanical strength of SPEs, thereby weakening the safety and cycling stability of cells. Another promising intermolecular strategy is blending polymer matrix with inorganic fillers, namely the inorganic/polymer hybrid solid electrolytes (IPHEs) [43–48]. Similarly, inorganic fillers disorder the crystallization of PEO and facilitate their segmental dynamics. More importantly, the interface between inorganic fillers and polymer matrix provides new pathways for fast ion transport, elevating the all-solid-state ionic conductivity up to 7.0 × 10^−4^ S cm^−1^, accompanied with a synchronous improvement of mechanical strength. The challenge of IPHEs is the difficulty in high-quality preparation, including manufacture craft of thin films [49–53] and homo-disperse of nanoparticles [54–56]. While recent state-of-the-art IPHE films have achieved thickness as thin as 20~30 μm [57, 58], most of current abilities are still 100~200 μm [59], translating to a high internal resistance and faded performance in practical pouch cells. The homo-disperse issues also exist in another widely studied hybrid route—physically blending PEO with their polymer homologues, such as poly(vinylidene fluoride) (PVDF), polyacrylonitrile (PAN) [60–64], in which the inherent thermodynamic incompatibility tends to drive phase separation [65, 66], eliciting spatial inhomogeneity in ion transport across electrolyte, and local polarization in batteries. Corresponding progresses in intermolecular strategies have been summarized in several previous reviews [67–69].

Distinct from material-level blending, intramolecular strategy focuses on the molecular-level redesign or modification of PEO matrix itself, representing a more fundamental route with greater potential to overcome the intrinsic limitations of PEO-based SPEs. Topologically, transforming ultra-long PEO backbones into short brushes [70–73], star-like [74–77], and even hyper branched architectures [78–80] can eliminate the crystallization, markedly accelerating segmental dynamics and ion transport. Mechanical strength can further be enhanced by block copolymerization [81–84], physical or chemical crosslinking strategies [85–88], granting PEO-based LMBs outstanding dendrite resistibility and cycling stability, even at high temperatures. Chemically, anchoring anions onto polymer backbones to fabricate a single-ion conductor can increase *t*_+_ close to 1, effectively stabilizing the space-charge field and Li^+^ electrodeposition. Additionally, judicious backbone or end group modifications to regulate highest occupied molecular orbital (HOMO) energy can also intrinsically expand the electrochemical stability window, enabling the compatibility with high-voltage cathode materials. Compared to intermolecular strategy, the bulk inhomogeneity and thin-film challenges caused by multi-phase blending are avoided readily. Additionally, intramolecular design of PEO itself would persistently perfect the polymer matrix, providing a more powerful platform that can further cooperate with intermolecular strategies, to fulfill more severe challenges in real battery application.

**Fig. 2:**
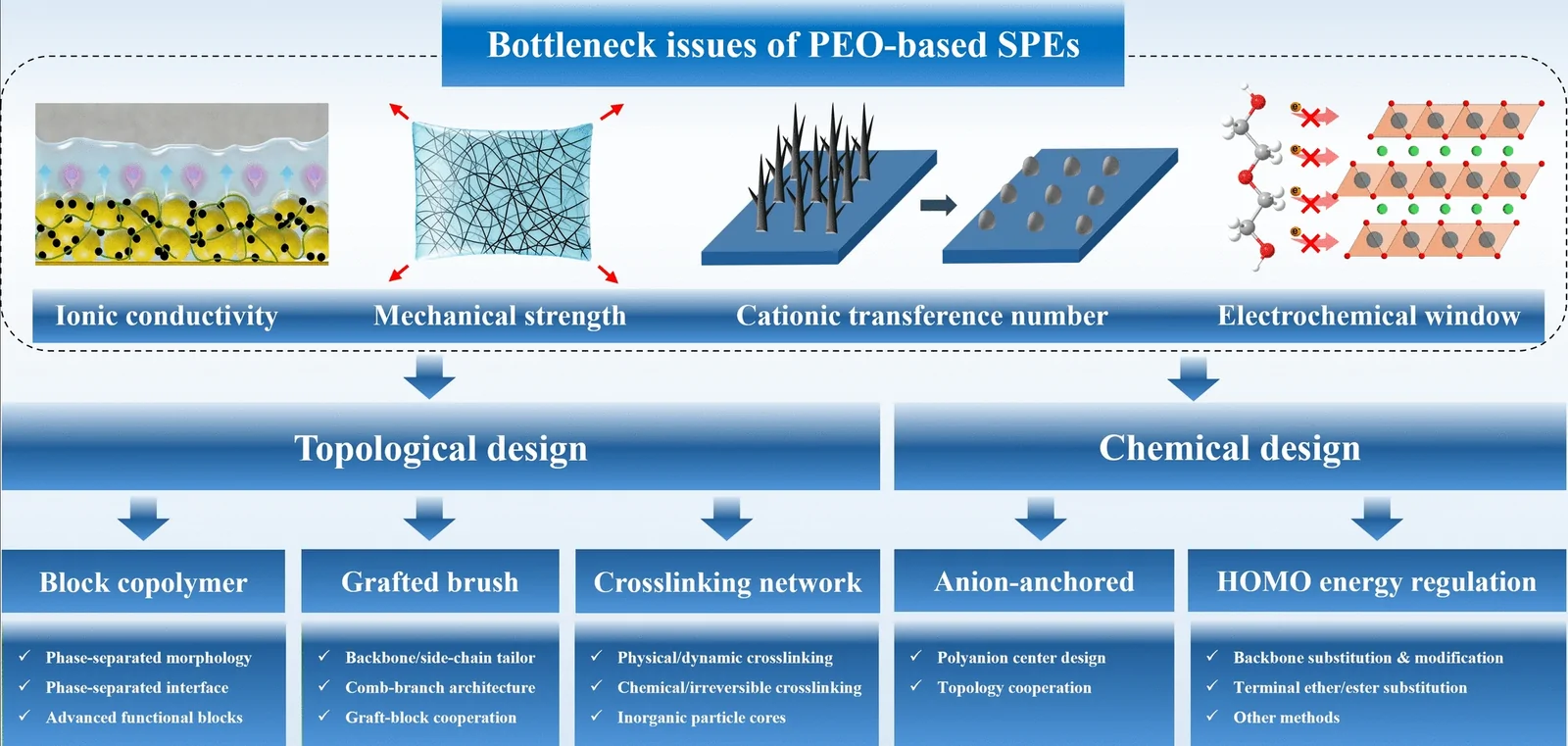
Schematics illustration of the issue-guided PEO-based SPEs intramolecular design strategies, which is categorized into two overarching directions—topology and chemistry

Despite profound summaries on PEO-based SPEs recently, existing reviews rarely decoupled PEO-based SPEs into intermolecular and intramolecular designs, tending to conflate the two and thereby failing to propose clear design principles. In fact, previous reviews predominantly centered on intermolecular approaches. Intramolecular strategy as critical yet independent direction, however, has not been systematically overviewed. Guided by the major bottlenecks of PEO-based SPEs, this work, for the first time, categorized intramolecular design of PEO into two overarching strategies: topology and chemistry (Fig. 2). Topological design primarily aims to resolve the inherent trade-off between ionic conductivity and mechanical strength, while chemical design focuses on addressing the low transference number and poor oxidation stability at high voltages. Following this framework, we connect the design methodology, structure–property relationship, and the underlying mechanisms organically, providing a comprehensive summary and a forward-looking perspective, which enlighten the exploration of more reliable SSEs and boost their practical application in next-generation high-energy batteries.

## Topological Design

### Block Copolymer Strategy

**Fig. 3:**
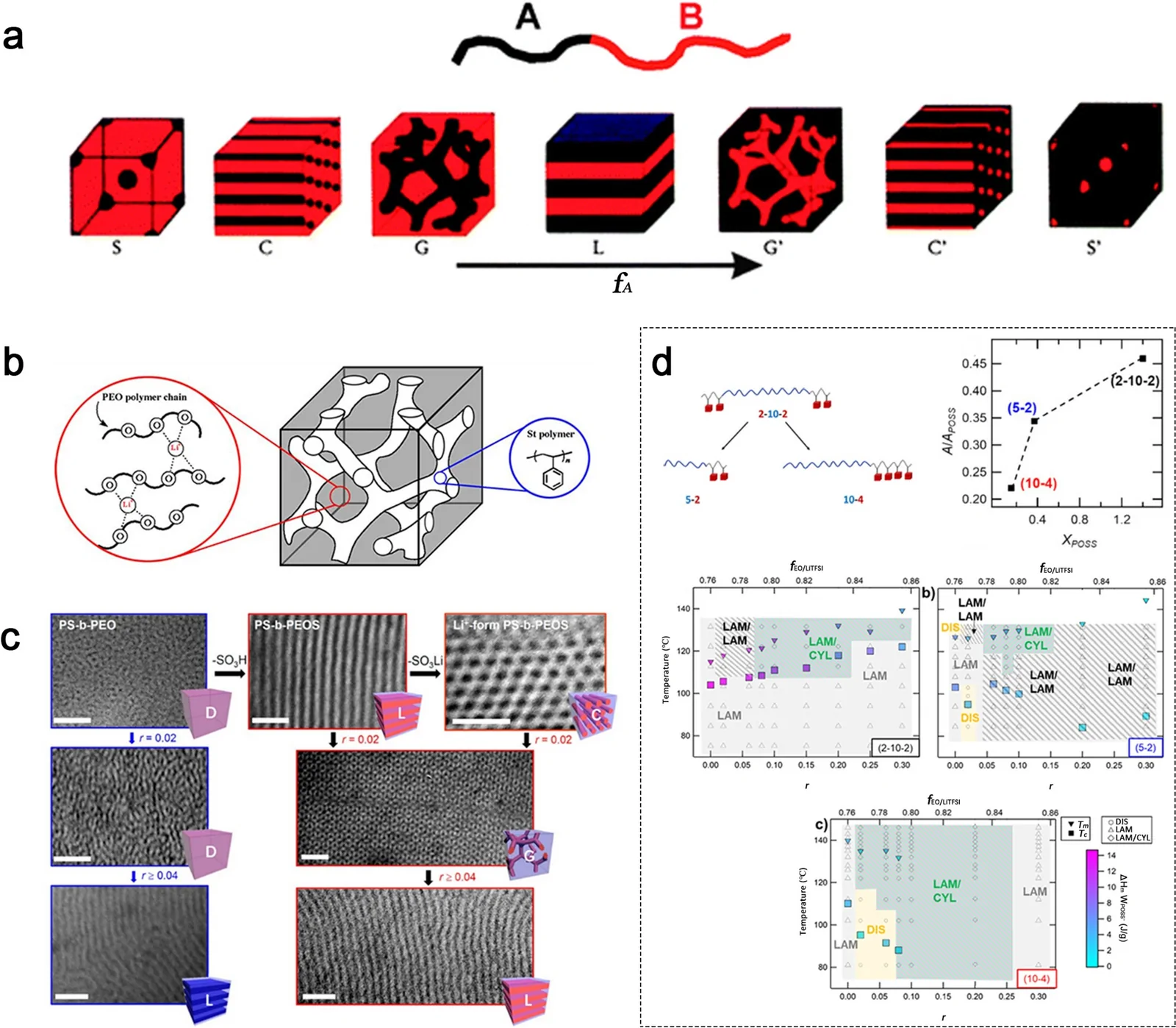
**a** Schematics of a di-block copolymer and its phase separation morphologies by self-assembly. **b** Schematic of solid PSt-b-PEO electrolytes (G morphology) [81]. Copyright 2005, Elsevier. **c** Effects of terminal group and salt concentration on nanophase separation behavior of PSt-b-PEO solid electrolytes [100]. Copyright 2013, American Chemical Society. **d** Chemical structure cartoons, percent crystallinity of the POSS domain (XPOSS), and phase diagrams of POSS_2_-*b*-PEO_10_-*b*-POSS_2_, PEO_5_-*b*-POSS_2_, and PEO_10_-*b*-POSS_4_ [101]. Copyright 2021, Elsevier

Block copolymers (BCPs) covalently link two or more polymer chains with distinct physicochemical natures and enable the formation of unique self-assembled structures, providing a powerful molecular engineering approach to enriching the functions and performance of SPEs [89–93]. Figure 3a shows a typical AB di-block copolymer and its self-assembled behavior. With volume fraction of block A (*f*_A_) increasing, the nanophase separation morphology transitions from body-centered cubic spherical (*S*) phase to cylindrical (*C*), double gyroid (*G*), lamellar (*L*) phases, and then inverted repeats [94, 95]. Existing insights in BCP science establish a steady foundation for the application of BCPs in SPEs [96]. For instance, combining flexible PEO with rigid segments is one effective strategy to address the trade-off between ionic conductivity and mechanical strength of SPEs. As depicted in Fig. 3b, PSt-*b*-PEO composed of polystyrene (PSt) and PEO blocks is one of the most classic matrices for BCP-based SPEs [81, 97, 98], in which the PEO segments provide rapid ion transport upon melting at temperatures above 60 °C and the “hard” PSt block possessing *T*_g_ over 100 °C offers adequate mechanical support, exhibiting modulus (10^7^–10^8^ Pa) two orders of magnitude higher than neat PEO [99].

#### Effect of Phase-Separated Morphology

Self-assembly of BCPs and their nanophase morphologies are critical to ion transport of electrolytes. In the past decade, impacts of molecular weight (*M*_n_), Li salt concentration, and end groups on phase separation behavior and ionic conductivity are intensely studied by Balsara, Park, and et al. [100, 102–104], utilizing PSt-*b*-PEO as a universal matrix. Generally, ionic conductivity increases with the *M*_n_ (*M*_PEO_) or volume fraction (*f*_PEO_) of PEO segments across all molar ratios of Li^+^ to ethylene oxide (EO) unit (*r*). Notably, a moderate Li salt concentration of *r* = 0.05~0.1 delivers the highest ionic conductivity. At a low Li salt loading, conductivity increases with *r* due to the increased dissociated charge carriers, whereas after a “saturation” threshold, further increasing Li^+^ reduces the PEO chains mobility through transient ion-segment crosslinking [105].

Apart from *r* and *f*, the phase-separated morphology shows a remarkable influence on ionic conductivity of BCP-based SPEs, as described in Eq. (1):

1 $$ \sigma_{{{\mathrm{BCP}}}} = Af_{c} \sigma_{c} $$

where *f*_*c*_ and *σ*_*c*_ are volume fraction and intrinsic conductivity of the conductive phase (PEO), respectively. Interestingly, the *A* is a morphology factor, which exhibits theoretical values of 1/3, 2/3, and 1 for *C*, *L*, and *G* morphologies, respectively. When *G* morphology is formed, the conductive PEO component creates a three-dimension continuous ion transport pathway and exhibits the highest ionic conductivity, whereas the *C* or *L* morphologies merely show one-dimension or two-dimension continuity, respectively [106]. As shown in Fig. 3c, changing end groups of PEO from –OH to –SO₃H and –SO₃Li can drive PSt-*b*-PEO to form various phase-separated morphologies from disorder (*D*) to *L* and *C* morphologies, respectively. When Li salts are blended, nanostructures of *D*, *L*, and *G* are further built, in which the *G*-phase SPE displays a twofold-enhanced ionic conductivity (~ 10^−4^ S cm^−1^ at 80 °C) than the others [100]. In addition to *f*, *r*, and even end group effect, Balsara et al. [101] recently find that the crystallizing behavior of non-conductive block also plays a key role in phase separation morphology of BCP-based SPEs (Fig. 3d). A series polyhedral oligomeric silsesquioxane (POSS)-*b*-PEO BCPs with identical *f*_POSS_ and *f*_PEO_ (POSS_2_-*b*-PEO_10_-*b*-POSS_2_, PEO_5_-*b*-POSS_2_, and PEO_10_-*b*-POSS_4_) are synthesized to exclude the *f* effect on nanophase morphology. They demonstrate the degree of polymerization (DP) and backbone sequence can influence the crystallization of POSS and further determines different nanophase morphologies.

#### Effect of Phase-Separated Interface

**Fig. 4:**
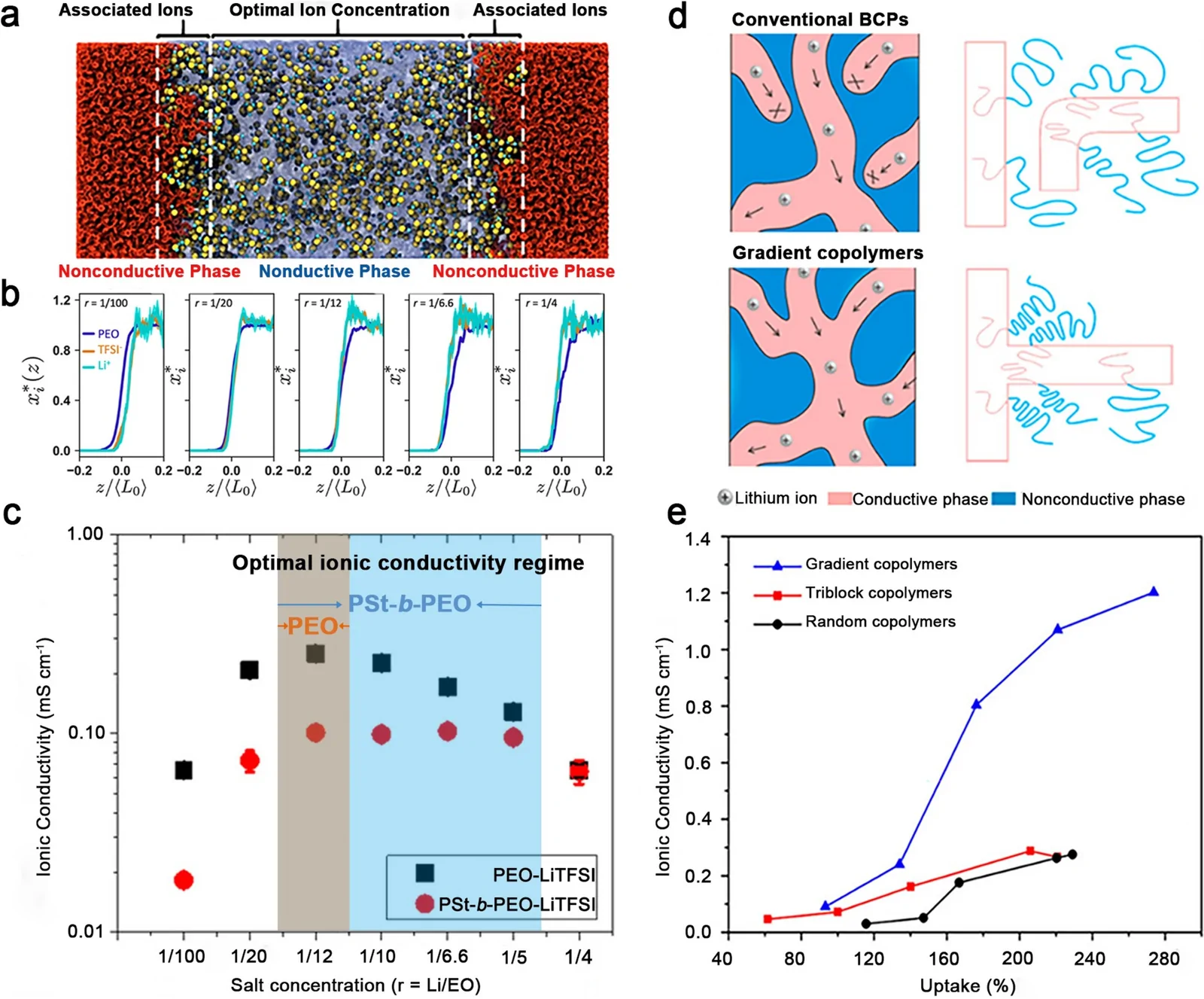
Effect of phase separation interface on ionic transport behavior. **a**–**c** BCPs phase-separated interface enables the optimal conductivity over a broad range of salt concentrations [97]. Copyright 2021, American Chemical Society. **a** Associated ion distribution within the conductive phase and at the phase-separated interface in PSt-*b*-PEO. **b** Normalized composition profiles, x_i_^*^, for PEO, TFSI^−^, and Li^+^ as a function of distance perpendicular to the PSt-*b*-PEO interface. **c** Ionic conductivity of PEO and PSt-*b*-PEO electrolytes at 100 °C as a function of LiTFSI concentration. **d**, **e** Gradient copolymers design alleviates the formation of discontinuous conductive pathways at the phase-separated interfaces [107]. Copyright 2016, American Chemical Society. **d** Schematics of discontinuous and continuous conducting pathways corresponding, respectively, to the morphological characteristics at the interface of conventional BCPs and gradient copolymers. **e** Ionic conductivity of GPEs based on conventional BCPs and gradient copolymers

Recently, unique thermodynamic nature at phase-separated interface is also determined as a key factor to regulate the ion transport behavior, albeit corresponding mechanism investigations are still insufficient. Nealey et al. [97] employed an interdigitated electrode to demonstrate that the phase-separated interface between the conductive and non-conductive domains in BCPs significantly influences associated ion distribution (Fig. 4a) and integrated it with molecular simulations to elucidate the differences in ion interactions and transport properties between PSt-*b*-PEO electrolytes and PEO homopolymers. The study demonstrated that, compared to PEO homopolymers, PSt-*b*-PEO can maintain the optimal value of conductivity over a wider range of Li salt concentrations (Fig. 4c). Molecular dynamics simulations reveal that, in PSt-*b*-PEO electrolytes, as the Li salt concentration gradually increases to a relatively high regimen (*r* > 1/12), the compositional distribution profile of PEO lags behind that of the salt species. This suggests a relatively higher *r* at the interface compared to the interior of the PEO domain (Fig. 4b). The phase-separated interface of the PSt-*b*-PEO electrolyte effectively sequestered “excess” Li salt, thereby preserving the optimal Li salt concentration within the PEO-based conductive phase and extending the tolerance range of optimal ionic conductivity with respect to Li salt concentration.

Nevertheless, compositional discontinuity at phase-separated interface often leads to defect structures. The thermodynamic repulsion forces between different segments can also reduce the mobility of the connecting segments, inhibiting the transport rate of Li^+^ at the phase boundaries [108–110]. In fact, conventional BCPs (especially *L* or *C* phases) composed of highly polar–nonpolar components exhibit strong phase separation and distinct domain boundary effects [111, 112]. The overlap of polymer chains around the junctions forces the blocks to contract to maintain a uniform segmental density distribution, and the entropic penalty drives the domains to bend at their boundaries. Consequently, nonpolar domains isolate some polar domains, forming numerous dead ends and cutting off polar pathways, which significantly impair ionic conduction. This effect is inevitable for BCPs with distinct polar–nonpolar characteristics. By preferentially introducing more reactive monomers and gradually adding inert monomers for gradient-growth polymerization, it is possible to obtain gradient BCPs with compositional transitions at the phase interfaces, providing a possibility to achieve BCP-based SPEs with minimal defects or grain boundaries after self-assembly [113]. Furthermore, the inter-segment repulsion in gradient copolymers is suppressed, allowing for a broader but moderate phase-separated interface [114], which offers a more continuous and smoother ion transport pathway [107, 115]. For instance, Gao et al. synthesized the gradient block copolymer PSt-*g*-poly(methyl acrylate) a matrix for GPEs (Fig. 4d). The reduced number of dead ends resulting from the gradient compositional change facilitates a more continuous ionic transport pathway, leading this electrolyte exhibit the highest ionic conductivity at RT compared to random copolymers and conventional triblock copolymers (Fig. 4e) [107]. Although the gradient copolymer strategy has not been applied in PEO system, above results indicate that a broader and moderate phase-separated interface could benefit for ionic conductivity, which could be a valuable research direction for PEO-based SPEs.

#### Exploration of Advanced Functional Blocks

While conventional rigid blocks such as PSt and POSS [101, 116, 117] enable efficient mechanical reinforcement for PEO-based SPEs, their application in practical solid-state batteries, still faces challenges. This is attributed to the severe yet comprehensive requirements for realistic battery operation. Therefore, in the past 5 years, various novel functional blocks as well as their coordination environment are developed in order to improve and balance the comprehensive properties, including electrochemical stability window (ESW), *t*_+_, and ionic conductivity.

**Fig. 5:**
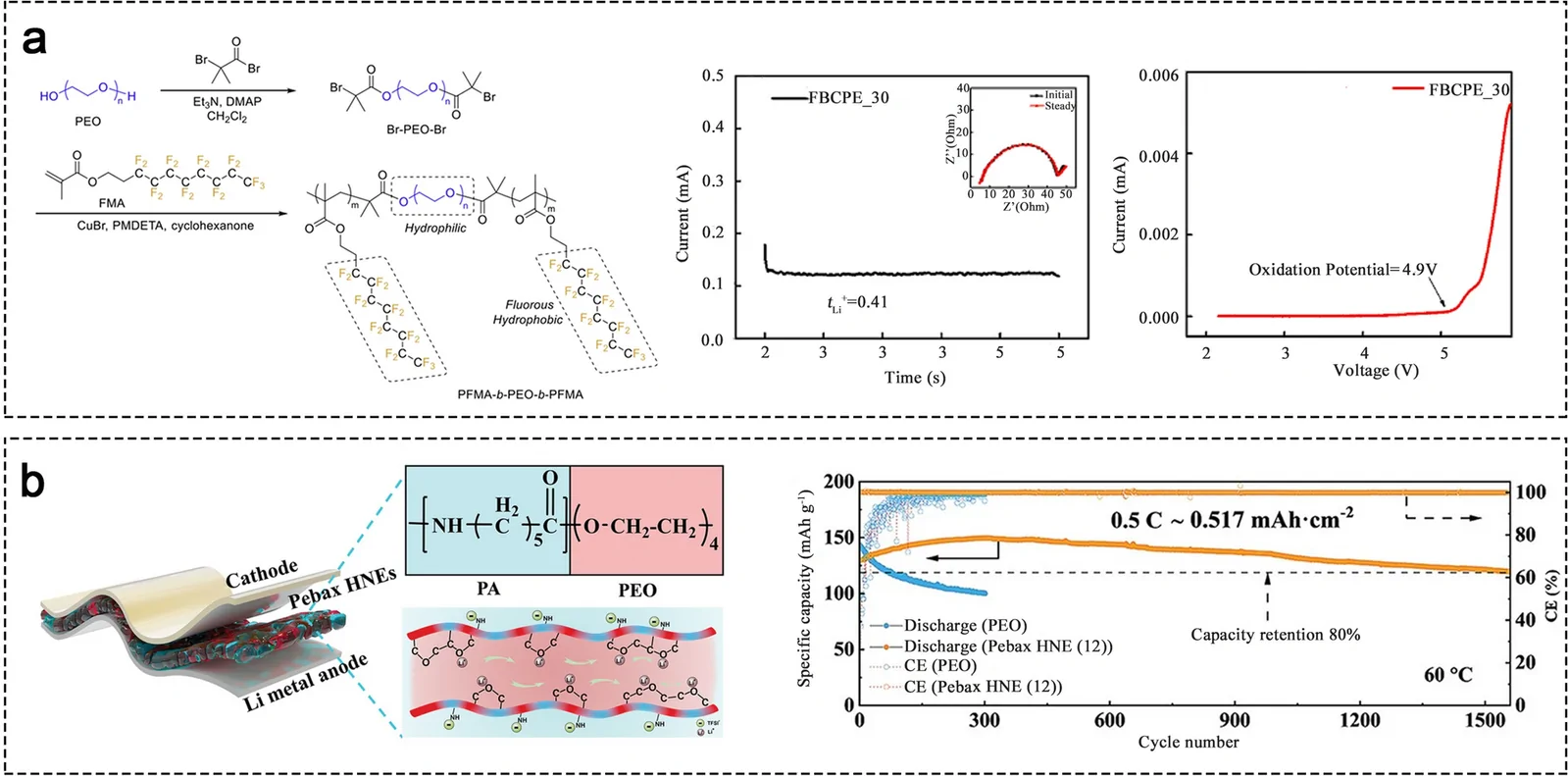
Advanced functional blocks are modified via fluorination and anion coordination strategies to enhance the antioxidant capacity, ionic conductivity, and mechanical properties of BCPs. **a** Synthesis route and linear sweep voltammetry (LSV) curves of PFMA-*b*-PEO-*b*-PFMA, as well as chronopotentiometry profiles of Li||Li symmetrical cell [118]. Copyright 2021, Elsevier. **b** Chemical structure, Li^+^ conduction mechanism of PA-*b*-PEO, along with the cycling performance in Li|PA-*b*-PEO|LFP cell [119]. Copyright 2022, John Wiley and Sons

Xie et al. [118] designed and synthesized a fluorinated ABA-type triblock copolymer solid electrolyte (PFMA-*b*-PEO-*b*-PFMA), while utilizing the fluorinated polymer electrolyte poly(heptadecafluorodecyl methacrylate) (PFMA) as the A block (Fig. 5a). The favorable fluorophilic interactions among fluorine-containing blocks enhance the mechanical properties (tensile strain: 440%) and *t*_+_ (0.41) of PFMA-*b*-PEO-*b*-PFMA. Furthermore, the lowering of the HOMO energy level across the entire block segment contributes to a broad ESW of up to 4.7 V (vs. Li^+^/Li). The battery fabricated with the electrolyte and LiNi_0.6_Co_0.2_Mn_0.2_O_2_ exhibits satisfactory cycling stability, maintaining 91.5% of its initial capacity after 20 cycles. In 2022, Wang et al. introduced a poly(ether-*block*-amide) (Pebax) strategy, as illustrated in Fig. 5b. This involved the preparation of a heterogeneous nanodomain electrolyte from rigid polyamide (PA) chains and flexible PEO chains, which exhibited a high ionic conductivity of 4 × 10^−4^ S cm^−1^ at 60 °C and appropriate mechanical strength (tensile strength: 6 MPa) [119]. The PA chains facilely coordinate with the anions of LiTFSI, which promotes rapid Li^+^ conduction and regulates uniform Li deposition. Additionally, the formation of a thin and dense solid electrolyte interphase (SEI) layer, along with the excellent mechanical strength of Pebax electrolytes, synergistically suppresses dendrite growth, enabling full solid-state LFP batteries to maintain a capacity retention of 80% after 1560 cycles at 0.5 C.

In spite of great progresses in comprehensive performance and battery application, RT ionic conductivity of BCP-based SPEs is still a formidable challenge due to the RT crystallization of PEO block. In this context, In 2024, Liu et al. [120] introduced a poly(lactic acid) (PLA) block into PSt-*b*-PEO architecture via sequential polymerization, creating a pentablock PSt-*b*-PLA-*b*-PEO-*b*-PLA-*b*-PSt SPE. Instead of PSt that exhibits strong incompatible with PEO, the intermediate PLA block shows excellent miscibility with PEO and form a PLA-PEO nano-mixed phase [121], which can effectively reduce PEO crystallinity. Also, the ester functionalities in PLA help to modulate Li^+^ coordination environment, facilitating a rapid Li^+^ transport. This strategy achieves a high ionic conductivity of 1.3 × 10^−4^ S cm^−1^ at RT and high tensile strength of 8.47 MPa, enabling an all-solid-state RT Li||LFP cell stably cycling at 0.1 C with 94.6% capacity retention after 200 cycles. Another successful case for improving RT ionic conductivity of BCP-based SPEs is based on a high-salt-concentration strategy. Kraus et al. [83, 84] found that in poly(isoprene) (PI)-*b*-PSt-*b*-PEO BCPs, even a small fraction of PEO blocks but a high Li salt concentrations (*r* = 4.30) can deliver ionic conductivity as high as 1.4 × 10^−3^ S cm^−1^ at 20 °C and a storage modulus (*G’*) of 10^7^ Pa. The highly ordered *L*-phase self-assembly morphology with small PEO channel established a beneficial “polymer-in-salt” environment for ion transport, which is decoupled from salt dissociation and polymer segmental motion [122]. Consequently, a high *t*_+_ of 0.7 is also achieved. However, RT battery performance is unreported, which might be attributed to the intrinsic interfacial wetting problem of high-salt-concentration system.

To date, researches on PEO-based SPEs via BCP strategies have continued nearly two decades, yielding a wealth of mechanistic insights and innovative results. Current findings indicate that linear BCP-based SPEs can effectively decouple the ionic conductivity and mechanical strength at elevated temperatures (≥ 60 °C), providing a powerful structural platform for high-temperature SSEs and all-solid-state batteries. Within 60–100 °C, these BCP electrolytes tend to achieve *G’* of 10^7^–10^8^ Pa and ionic conductivity of 10^−4^–10^−3^ S cm^−1^. Typical data are summarized in Table 1. However, due to the inherent limitation of linear structures, which fails to alter the high crystallinity and slow mobility of PEO segments at RT, room-temperature applications are still challenging. Combination of flexible blocks, such as polydimethylsiloxane (PDMS) and polyethylene (PE) with PEO, can somehow enhance segmental motion [123, 124], but the RT ionic conductivity remains 10^−6^ to 10^−5^ S cm^−1^ only. Therefore, to enhance the RT ionic conductivity of PEO-based SPEs, the design of topological structures with faster RT dynamics will be of great significance.

**Table 1:** Performance comparison of PEO electrolytes based on topological design

Entry	Matrix	Ionic conductivity (S cm^−1^)	Modulus (MPa)	*E*_ox_ (V vs. Li/Li^+^)	Cycling performance	Electrode materials	References
*Block copolymer strategy*							
1	PSt^a)^-*b*-PEO	~ 2 × 10^−4^ (90 °C)	50	–	–	–	[82, 125, 126]
2	POSS^b)^-*b*-PEO	~ 10^−7^ (30 °C)	–	4.6	–	–	[127, 128]
		~ 10^−6^ (90 °C)					
3	PA^c)^-*b*-PEO	4 × 10^−4^ (60 °C)	6	4.38	0.5 C; 1560th (60 °C); Solid	Li||LFP	[119]
4	PFMA^d)^-*b*-PEO-*b*-PFMA	2.68 × 10^−4^ (70 °C)	–	4.9	0.2 C; 250th (70 °C); Solid	Li||LFP	[118]
5	PSt-*b*-PLA^e)^-*b*-PEO-*b*-PLA-*b*-PSt	1.3 × 10^−4^ (RT)	8.47	4.2	0.1 C; 200th (RT); Solid	Li||LFP	[120]
6	PI^f)^-*b*-PSt-*b*-PEO	1.4 × 10^−3^ (20 °C)	10	–	–	–	[84]
*Side-chain grafted strategy*							
7	PC^g)^-*g*PEG	2.0 × 10^−5^ (30 °C)	1.18	5.5	0.5 C; 200th (60 °C); Solid	Li||LFP	[129]
8	Crown ether PEO	1.48 × 10^−5^ (20 °C)	–	5.3	0.1 C; 150th (60 °C); Solid	Li||LFP	[75]
9	PSt-*b*-PPEGMA^h)^-*b*-PSt	2 × 10^−4^ (30 °C)	3	4.5	0.1 C; 100th (30 °C); Solid	Li|| lithium cobalt oxide (LCO)	[81]
10	Boronic ester-based polymers	2.2 × 10^−4^ (60 °C)	–	4.7	0.2 C; 150th (60 °C); Solid	Li||LFP	[130]
11	PAALi-*g*PPEGMA-gPTMC-*co-*TFEMA^i)^	2.46 × 10^−4^ (30 °C)	–	5.3	2 C, 3 C; 1000th (60 °C); Solid	Li||LFP	[131]
12	*g*PSt-*b*-*g*PEO-*b*-*g*PSt	1 × 10^−3^ (105 °C)	1 × 10^−2^	–	–	–	[132]
13	*g*PMPCS^j)^-*b*-*g*PEO	1.58 × 10^−3^ (200 °C)	–	–	–	–	[133]
14	*g*PDMS^k)^-*b*-*g*PEO	2.0 × 10^−4^ (RT)	~ 0.1	–	–	–	[105]
15	EHPI^l)^-gPEG	7.12 × 10^−4^ (RT)		> 4.5	0.5 C; 550th (RT); Solid	Li||LFP	[134]
16	PPMALi^m)^-*g*PEG	4.49 × 10^−5^ (30 °C)	2.60	4.48	0.1 C; 100th (60 °C); Solid	Li||LFP	[70]
17	Star polymers with comb-like arms	6.14 × 10^−5^ (30 °C)	~ 10^−3^	–	–	–	[76]
18	*hb*PS-*star*-PPEGMA^n)^	9.5 × 10^−5^ (60 °C)	1.4	4.34	0.2 C; 100th (60 °C); Solid	Li||LFP	[78]
*Crosslinking strategy*							
19	PPO-*b*-PEO-*b*-PPO-*c*-UPy^o)^	1.2 × 10^−4^ (RT)	14	–	0.2 C; 400th (25 °C); Quasi-solid	LTO||LFP	[135]
20	CuF_2_-*c*- PEO	2 × 10^−4^ (30 °C)	57.3	4.6	0.6 C; 500th (30 °C); Solid	Li||NCM	[136]
21	PEO-*c*-boric	3.5 × 10^−4^ (90 °C)	1–10	–	–	–	[137]
22	PGMA-*c*-PEG^p)^	1.31 × 10^−4^ (40 °C)	3.4	5.3	0.1 C; 140th (RT); Solid	Li||LFP	[138]
23	POSS-*c*-*g*PEO	2.3 × 10^−4^ (RT)	57	–	0.1 mA cm^−2^; 1000 h (RT); Solid	Li||Li	[139]
24	PE^q)^-*c*-PEO	> 1.0 × 10^−4^ (RT)	0.1	–	–	–	[87]
25	LA-*c*-PAM-*c*-PEO^r)^	6.1 × 10^−4^ (60 °C)	7.83	4.95	1 C; 1000th (60 °C); Solid	Li||LFP	[140]
26	PEO-*c*-TEGDME-*c*-PAN^s)^	1.72 × 10^−3^ (RT)	–	4.6	0.5 C; 150th (RT ℃); Solid	Li||NCM622	[141]
27	Uio-66-*c*-PETMP-*c*-PEGDA^t)^	2.26 × 10^−4^ (RT)	9.4	5.4	0.5 C; 500th (40 °C); Solid	Li||LFP	[142]
28	PEO-*c*-C1^u)^	5.8 × 10^−5^ (30 °C)	0.595	5.21	0.5 C; 400th (60 °C; Solid	Li||LFP	[143]
29	HMDI-*b*-PEG-*b*-HMDI-*c*-ZrMOF^v)^	5.7 × 10^−4^ (30 °C)	76.5	5.1	0.3 C; 1000th (30 °C); Solid	Li||LFP	[144]

a) *PSt* polystyrene; b) *POSS* polyhedral oligomeric silsesquioxane; c) *PA* polyamide; d) *PFMA* poly(3,3,4,4,5,5,6,6,7,7,8,8,9,9,10,10-heptadecafluorodecyl methacrylate); e) *PLA* poly(lactic acid); f) *PI* poly(isoprene); g) *PC* polycarbonates; h) *PPEGMA* poly(poly(ethylene glycol) methyl ether methacrylate); i) *PAALi* lithium polyacrylate, PTMC = poly(trimethylene carbonate), TFEMA = trifluoroethyl methacrylate; j) *PMPCS* poly(2,5-bis((4-methoxyphenyl)oxycarbonyl)styrene); k) *PDMS* polydimethylsiloxane; l) *EHPI* 3,6-epoxy-n-hydroxy-1,2,3,6-tetrahydrophthalimide; m) *PPMALi* poly 2-((propionyloxy)methyl) lithium acrylate; n) star polymers with hyperbranched PSt core and poly[poly(ethylene glycol) methyl ether methacrylate] arms; o) *PPO* poly(propylene oxide), UPy = 2-ureido-4-pyrimidone; p) poly(glycidyl methacrylate)-crosslinked poly(ethylene glycol); q) *PE* polyethylene; r) lithium alginate-crosslinked polyethylene oxide-crosslinked polyacrylamide; s) poly(ethylene oxide)-tetraethylene glycol dimethyl ether-polyacrylonitrile; t) Uio-66-crosslinked tetrakis(3-mercaptopropionic acid) pentaerythritol-crosslinked poly(ethylene glycol) diacrylate; u) *C1* 3,3′-((perfluoropropane-2,2-diyl)-bis(4,1-phenylene))bis(3-(trifluoromethyl)-3H-diazirine); v) *HMDI* 4,4′-methylenebis(cyclohexyl isocyanate)

### Side-Chain Grafted Strategy

Comb or brush-like grafted polymers, consisting of a polymer backbone with densely packed side chains, allow for flexible tunability of backbone or side-chain composition and delivering unique advantages in application of PEO-based SPEs [145–148]. On the one hand, side-chain grafted strategy can suppress or even eliminate the crystallization associated with linear PEO polymer chains and enhance RT ionic conductivity. On the other hand, valuable functional groups can be easily introduced into the brush-like architectures by integrating BCP topologies or anion-anchored strategy, improving the comprehensive performance required by real battery applications.

#### Backbone and Side-Chain Chemistry

**Fig. 6:**
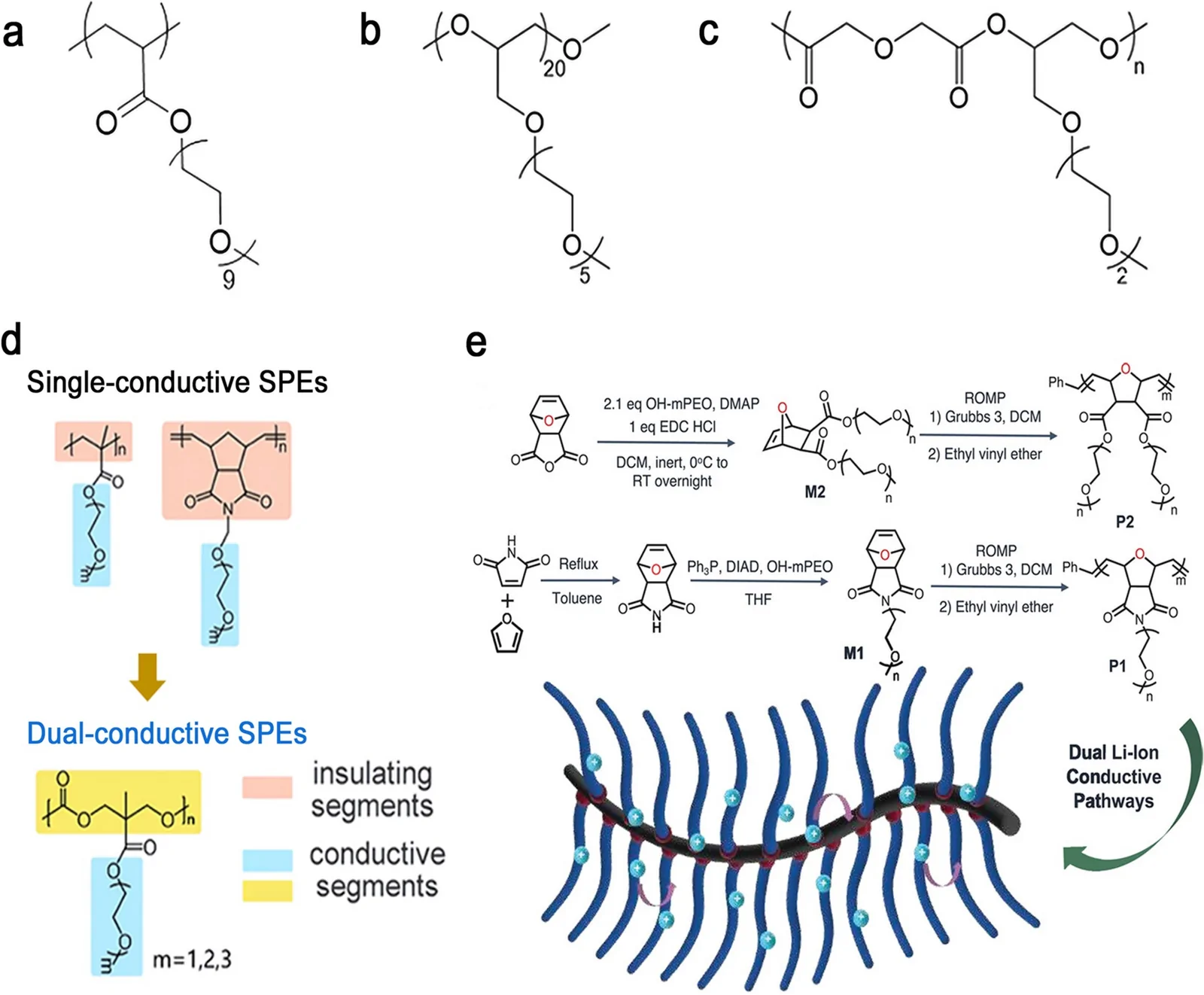
**a**–**c** Side-chain grafted SPEs based on various backbones and PEO side chain. **d**, **e** Strategies of constructing dual-Li⁺ conduction pathways: **d** schematics showing the strategy by transforming the insulating alkyl backbones to conductive PC backbones [129]. Copyright 2024, John Wiley and Sons. **e** Synthetic route of dual-Li⁺ conductive brush-like SPEs based on polyoxanorbornene backbone and cartoon showing the dual-Li^+^ transport mechanism [134]. Copyright 2023, John Wiley and Sons

Bannister et al. [73] initiated researches on side-chain grafted SPEs by employing low-*M*_n_ PEO-methacrylate as a macromolecular monomer, establishing a brush-like fast ion conductors composed of short PEO side chains (Fig. 6a). While this architecture exhibits similar ionic conductivity to conventional linear PEO at high temperatures, surprisingly, their RT ionic conductivity can achieve 2.1 × 10^−5^ S cm^−1^, which is two orders of magnitude higher than that of conventional PEO electrolytes [149]. This enhancement is contributed by two mechanisms. Firstly, the crystallinity of PEO is decreasing with chain length. Once the *M*_n_ of linear PEO is declined to 2000~100 Da, the mobility of PEO chains will be remarked promoted, and the state gradually transforms from semi-crystalline to amorphous. Secondly, when such short chains serve as side chains covalently linked to a backbone, the linking nodes can further prevent EO segments from packing into ordered crystalline structures. As a result, the brush-like topology with side chains consisting of 9 EO units (~ 400 Da) exhibits high mobility and ionic conductivity at RT.

Since then, various side-chain grafted SPEs based on different backbone and side-chain chemistries have been synthesized and applied as SPEs (Fig. 6b, c) [134, 150–154]. However, the relatively low ionic conductivity at RT continues to represent a significant challenge impeding the practical implementation of brush-type SPEs. Traditional graft polymers are mostly prepared via free radical polymerization (e.g., vinyl monomers) or ring-opening metathesis polymerization (ROMP, e.g., norbornene-based monomers), feature-insulating backbones that reduce the overall Li^+^ conductivity. Recently, Wen et al. [129] designed and synthesized a series of polycarbonates (PCs) with EO side chains via ROP and evaluated their ionic conductivities as SPEs when combined with LiTFSI (Fig. 6d). The synergistic effect of the conductive backbone and side chains enabled the *t*_+_ value to reach 0.67, while the ionic conductivity was 2 × 10^−5^ S cm^−1^ at 30 °C. This dual-conductive graft polymer simultaneously provides favorable Li^+^ conductivity and a high *t*_+_ value, enabling the Li||LFP battery to exhibit excellent cycling performance at 0.3 C (30 °C) and 0.5 C (60 °C). Matyjaszewski et al. [134] introduced ether oxygen groups into the backbone via ROMP, developing polyoxanorbornene-based bottlebrush polymers (P1, P2) with two distinct PEO side-chain linkage configurations (Fig. 6e). Beyond the ion-conductive PEO side chains, the polymer backbone incorporates additional ion-conducting moieties that facilitate Li^+^ transport within the SPEs matrix, enabling a high RT ionic conductivity of 7.12 × 10^−4^ S cm^−1^.

**Fig. 7:**
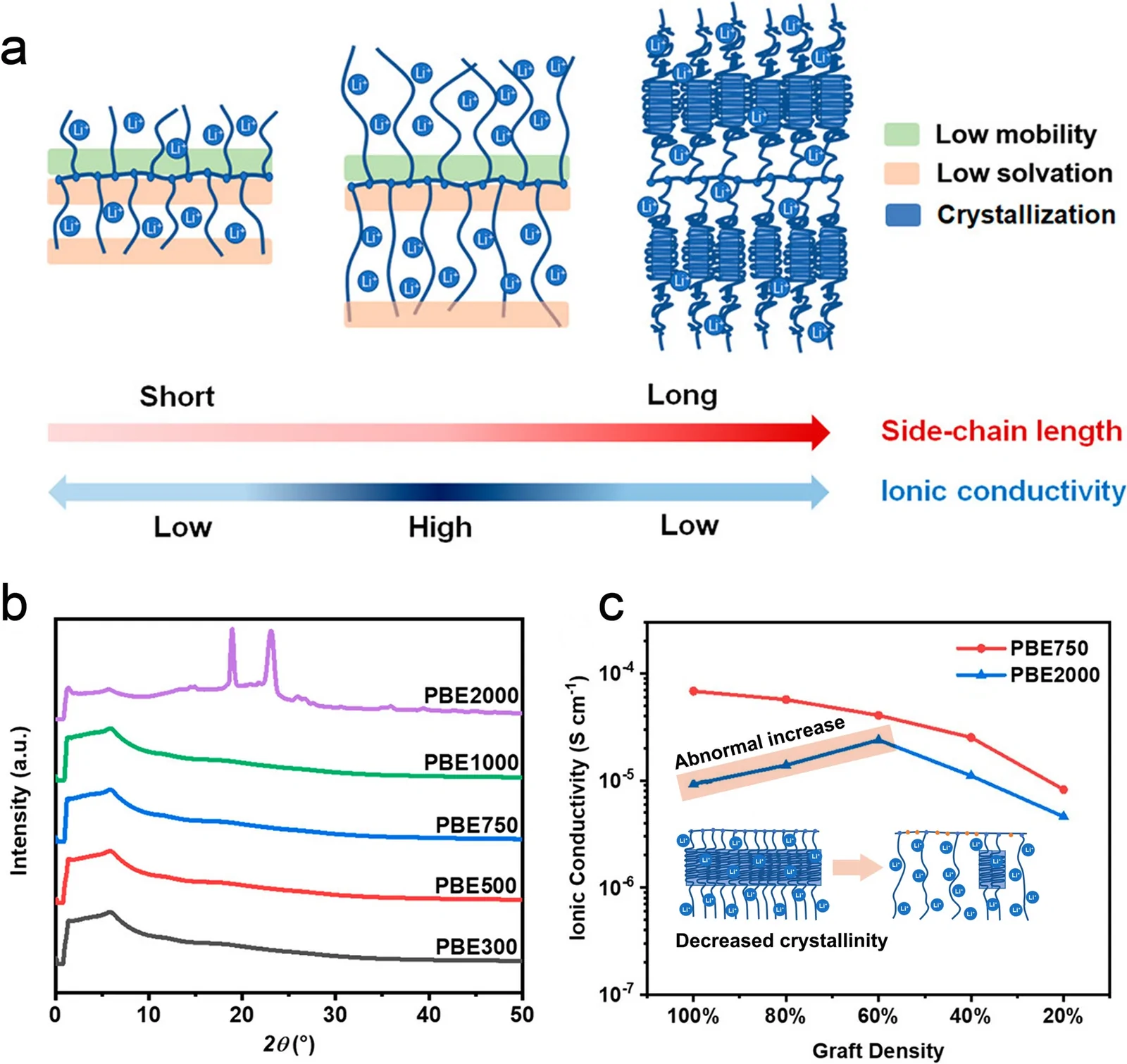
Comprehensive elucidation of the structure–property relationship between ionic conductivity and side-chain architectures. **a** Schematics showing a firstly increasing and then-decreasing tendency of ionic conductivity with side-chain length increasing, based on a competitive mechanism between heterogeneous distribution of solvation probability and segmental mobility, and crystallization. **b** WAXD (room-temperature) profiles at various side-chain lengths. **c** Ionic conductivity variation with graft density from 100% to 20% at different side-chain length regimes (*M*_*n*_ = 300, 750, and 2000 Da) and the corresponding schematic showing the crystallinity decline with graft density at side-chain length of 2000 Da [156]. Copyright 2022, American Chemical Society

Beyond backbone architecture, ion transport behavior is also governed by the side-chain architectures, notably their length and graft density. Early studies based on side-chain grafted polymers with 1–3 [125] or 1–9 [155] EO units suggest that a longer side chain can deliver a higher ionic conductivity. However, a side-chain length below 9 EO units (*M*_n_ < 400 Da) is limited, failing to comprehensively reveal the effect mechanism of side-chain length and precisely guide the rational design of side-chain grafted SPEs. In 2022, our group carried out a systematic study on the influence of side-chain architectures including both length and density dimensions. Meanwhile, the side-chain length is expanded to ~ 44 EO units (*M*_n_ = 2000 Da), encompassing both amorphous and crystalline regimes of PEO [156]. It is found that below ~ 17 EO units (~ 750 Da), RT ionic conductivity continuously increases with the side-chain length. The underlying mechanism is shown in Fig. 7a. Since the mobility of the side-chain roots is remarkedly restrained by the linked backbones, they contribute little in ion transport. Moreover, theoretical calculation demonstrates that the side-chain roots exhibit poor Li salt dissociation, also indicating a disability to ion transport [157]. With side-chain length rising, the ratio and effect of these “frozen” segments are gradually decrease, thereby showing an increasing ionic conductivity. However, with side-chain length further extending from 1000 into 2000 Da, thermodynamics experiments such as wide-angle X-ray diffraction (WAXD) suggest that entanglement and crystallization of the side chains occur (Fig. 7b), leading to a decrease of ionic conductivity. Consequently, intermediate length of ~ 750 Da yields the maximum ionic conductivity.

With regard to graft density, crystallization feature associated with side-chain *M*_n_ also plays a key role in the ion transport behavior. Within amorphous regime (*M*_n_ of side chain below ~ 750 Da), ionic conductivity increases with graft density and achieves the highest at 100% graft density (Fig. 7c). This can be attributed to higher connectivity of the Li^+^ coordination sites (oxygen atoms on PEO segments) with the increased graft density, which facilitates the ion hopping among sites. Within crystalline regime (such as side-chain* M*_n_ of 2000 Da), however, ionic conductivity only increases with graft density from 20% to 60%, after which the ionic conductivity begins to decrease. The mechanism of this unusual reduction is revealed by crystallinity calculation from differential scanning calorimetry (DSC). Surprisingly, the crystallinity of PBE2000 continuously increases from 2.1% to 10.7% with graft density from 20 to 100%. At graft density beyond 60%, the enhanced crystallization limits the segmental relaxation (inset of Fig. 7c), dominating the effect on ionic conductivity and leading to its decline.

#### Comb-Branch Architecture

**Fig. 8:**
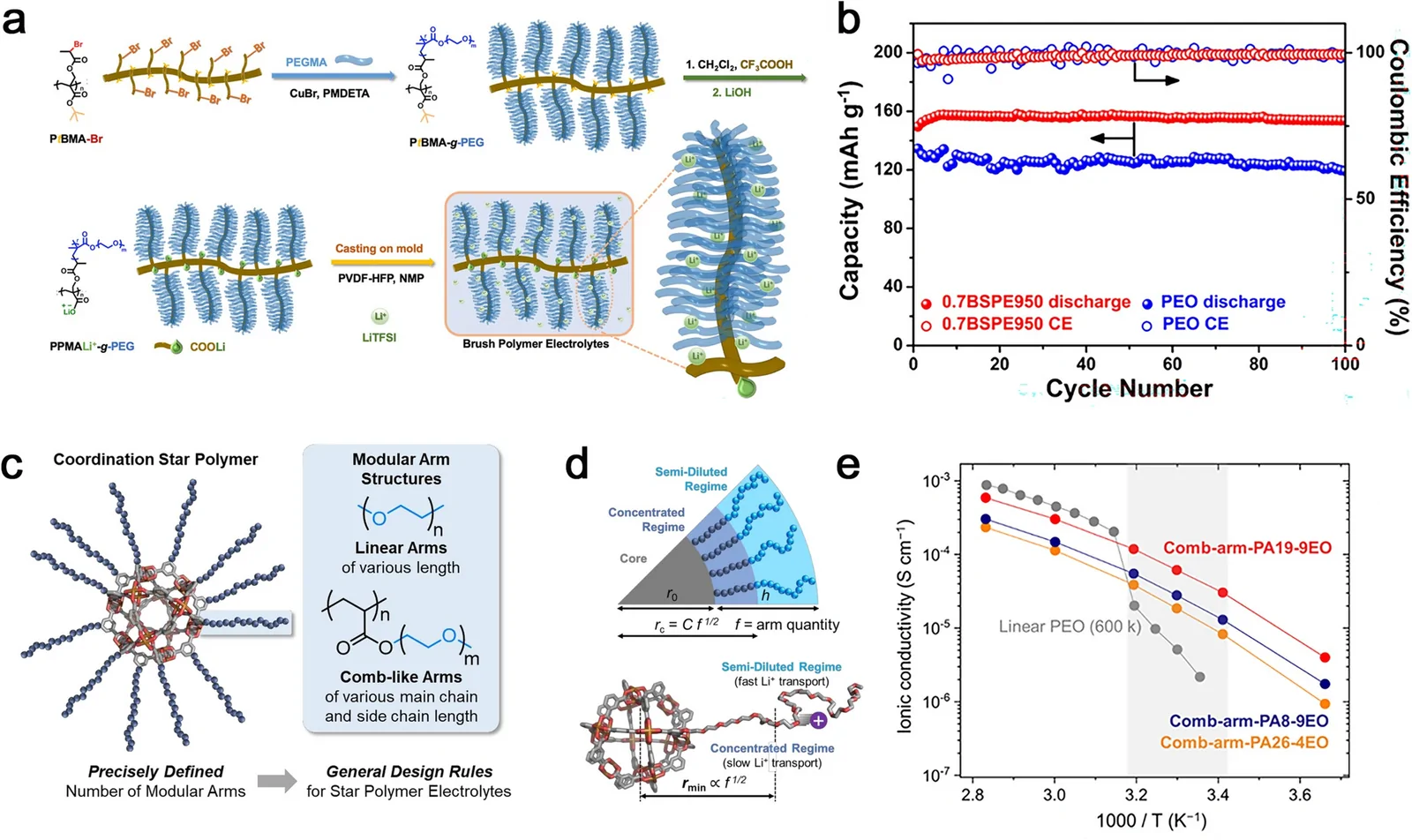
SPEs with various topological architectures featuring PEO comb-like branches. **a** Schematic diagram of highly branched comb-like PPMALi-g-PEG and **b** its corresponding Li||LFP cycling performance [70]. Copyright 2019, American Chemical Society. **c**–**e** Design and ion conduction mechanism of the comb-like arm coordination star polymer electrolyte [76]. Copyright 2025, chemrxiv: **c** Schematic illustration of the polymer structure. **d** Mechanism model explaining the high ionic conductivity. **e** Ionic conductivity plot

Branched comb-like architectures featuring appropriately dense PEO side chains are essential for enhancing the ionic conductivity of PEO-based SPEs. In 2019, Xue et al. [70] employed controlled radical polymerization techniques such as reversible addition-fragmentation chain transfer (RAFT) and atom transfer radical polymerization (ATRP) to design and synthesize efficiently a series of precisely structured grafted SPEs based on molecular bottlebrush with low polydispersity (PPMALi-*g*-PEG) (Fig. 8a). These brush macromolecules were connected to Li^+^, facilitating Li^+^ transport and increasing the *t*_+_. They exhibited an ionic conductivity of 4.5 × 10^−5^ S cm^−1^ and a *t*_+_ of 0.6 at RT. Furthermore, the Li||LFP batteries assembled with this electrolyte demonstrated high capacity and cycling performance at 60 °C (Fig. 8b), indicating that grafted brush SPEs with high branching structures are promising candidate materials for high-performance LMBs.

Star-shaped PEO polymers with a core and multiple polymer arms have been proven to be effective for enhancing RT electrical conductivity [158, 159]. However, there is still a lack of clear structure–property relationships to guide the design and optimization of star polymer electrolyte materials. In 2025, Qiao et al. [76] employ an alternative synthetic strategy to systematically vary the arm structures (linear arm, comb-like arms) of a series of star polymers (Fig. 8c). Building on the Daoud and Cotton model, they proposed that star-like polymers with linear arms have an intermediate and optimal arm length balance between the concentrated regime and the semi-diluted regime in two proportions to support efficient Li^+^ conduction (Fig. 8d). Their findings further revealed that comb-like arms are more effective than linear ones in reducing the crystallinity of star polymers and enhancing ionic conductivity. Specifically, higher DP of the main chain and longer comb side chains were identified as key factors leading to higher ionic conductivities in comb-arm star polymers (Fig. 8e).

#### Graft Block Cooperation

**Fig. 9:**
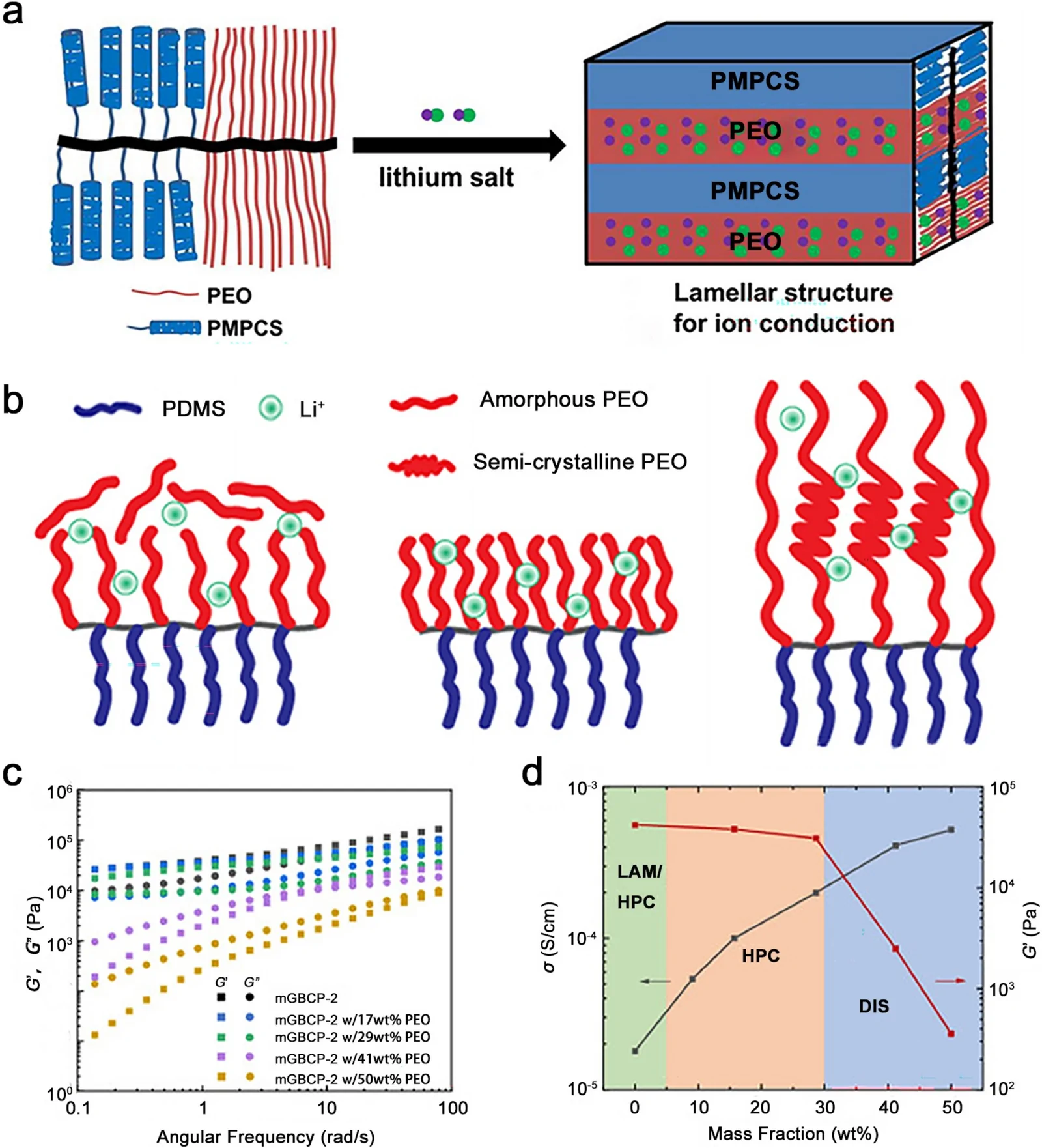
Topological approaches combining grafting and block strategies. **a** Molecular schematics and morphology of *g*PMPCS-*b*-*g*PEO [133]. Copyright 2017, American Chemical Society. **b** Schematic illustrations of mGBCP-based solid electrolytes based on PDMS and PEO side chains. **c** Rheological properties of mGBCP electrolytes with varying free PEO content. **d** Relationship of conductivity, phase separation morphology, and *G’* as a function of PEO fraction [105]. Copyright 2020, Elsevier

The migration of Li^+^ relies on the segmental relaxation motion of polymer chains. Although grafted polymer electrolytes with short brush-like side chains exhibit enhanced segmental mobility, the intrinsic flexibility of the brush molecules results in lower mechanical strength [160, 161]. Conversely, while traditional linear BCPs possess higher mechanical strength, they require larger block molecular weights and high temperatures to achieve sufficient ionic mobility, resulting in inadequate RT ionic conductivity. Therefore, cooperation of graft and block strategies becomes a competitive topological method to integrate their merits. For instance, Grubbs and Fan et al. utilized the ROMP of norbornene to copolymerize PSt monomers [132] and chiral liquid crystalline poly(2,5-bis((4-methoxyphenyl)oxycarbonyl)styrene) (PMPCS) monomers [133] with PEO, thereby obtaining grafted BCPs such as *g*PSt-*b*-*g*PEO-*b*-*g*PSt and *g*PMPCS-*b*-*g*PEO (Fig. 9a). Upon blending with Li salts, the *g*PMPCS-*b*-*g*PEO and *g*PSt-*b*-*g*PEO-*b*-*g*PSt electrolytes self-assembled into *L* and *C* phase structures, maintaining ionic conductivities around 2.0 × 10^−5^ S cm^−1^ while achieving *G’* on the order of 10^5^ Pa.

Recently, our group designed a class of SSEs based on mixed-graft block copolymer (mGBCP) architectures containing short PEO and PDMS side chains, as depicted in Fig. 9b [105]. The strong thermodynamic immiscibility between PEO and PDMS combining with backbone-suppressed mixing entropy resulted in the phase-separated nanostructure of ultra-low *M*_n_ of PEO chains (400~750 Da), which is confirmed to be a vital factor to maintain a high mechanical strength. Moreover, the short PEO side chains instead to long PEO chains in conventional BCPs can provide higher segmental mobility and ionic conductivity. Within *C* phase-separated morphology, such short PEO side-chain design with controlled blending of free PEO short chains (< 30 wt%) achieved the first all-solid-state PEO-based SPEs with RT conductivity of up to 2.0 × 10^−4^ S cm^−1^, yet without sacrifice of mechanical strength (10^4^–10^5^ Pa in *G’*) (Fig. 9c, d).

With rapid development of polymerization techniques, in the past decade, researchers created side-chain grafted SPEs with various brush-like architectures and applied them in high-energy solid-state LMBs. Typical data are summarized in Table 1. Overall, side-chain grafted strategy exhibits the most promising potential to fulfill eligible RT ionic conductivities (over 10^−4^ S cm^−1^) in a dry polymer all-solid-state condition. Backbone chemistry and side-chain topologies are two key factors to capture the highest ionic conductivity, by means of constructing dual-Li^+^ conductive pathways and attaining the fastest segmental mobility, respectively. As brush-like macromolecules tend to show a viscoelastic property and thereby a low mechanical strength, mGBCP strategies become predominant and valuable, despite still 1~2 orders of magnitude lower than linear BCP-based SPEs. Enhancing backbone rigidity while maintaining the high mobility of short side chains, through more ingenious intramolecular methods such as backbone crosslinking, can be further developed. Also, some advanced brush-like SPEs rely on complex and stringent synthetic condition, translating to a difficulty in scale-up production and film-fabricating process—factors that warrant consideration as well.

Additionally, while Table 1 summarizes and demonstrates the key performance of typical work, it is found that critical parameters are often missing, such as full-cell performance, or not normalized at identical condition, including operating temperature and *C*-rate. The loss of critical property and standardized metrics makes it difficult to evaluate the “advancedness” among different design methods. Therefore, we propose a set of core test items that must be contained in each work (Table 2), no matter in main text or supporting document. We believe this undoubtedly promotes fair comparison and elevates the researching efficiency, playing a key role in accelerating high-energy batteries from lab-scale research to industrialization.

**Table 2:** Standardized metric checklist of PEO-based SPE for efficient community evaluation

Entry	Evaluation item	Normalized test condition
1	Ionic conductivity	Electrochemical impedance spectroscopy at RT (20~30 °C)
2	Cationic transference number	Bruce–Vincent–Evans method with symmetric Li||SPE||Li cells at 0.1~0.2 mA cm^−2^
3	Electrochemical stability window	Linear sweep voltammetry or cyclic voltammetry at a scan rate of 1 mV s^−1^
4	Full-cell performance	Matching with Li anode and high-voltage cathode such as NCM to achieve cutoff voltage over 4.3 V
5	Cycling performance	Galvanostatic charge/discharge cycling at 0.1 C/0.1 C and 0.1 C/0.5 C at RT
6	Storage modulus*	Rheological oscillating sweeping result at frequency of 1 Hz and RT

^*^If mechanical strength of the designed SPEs is emphasized

### Crosslinking Strategy

Crosslinking PEO backbone to form three-dimensional (3D) network not only suppresses the PEO crystallization but also significantly enhances the molecular rigidity of SPEs matrix, making it an intensely studied topology for SPEs. Based on the nature of crosslinking interactions, it can be classified into two major categories: dynamic crosslinking and chemical crosslinking. Dynamic crosslinking employs physical or reversible interactions, including hydrogen bond, metal ion coordination, and dynamic covalent bond, endowing SPEs specific self-healing, stimulus responsiveness, and shape-memory properties. In contrast, chemical crosslinking is robust yet irreversible through permanent chemical bonds, which is being precisely designed and widely used in solid-state LMBs due to its outstanding mechanical strength and dendrites resistibility.

#### Physical or Dynamic Crosslinking

**Fig. 10:**
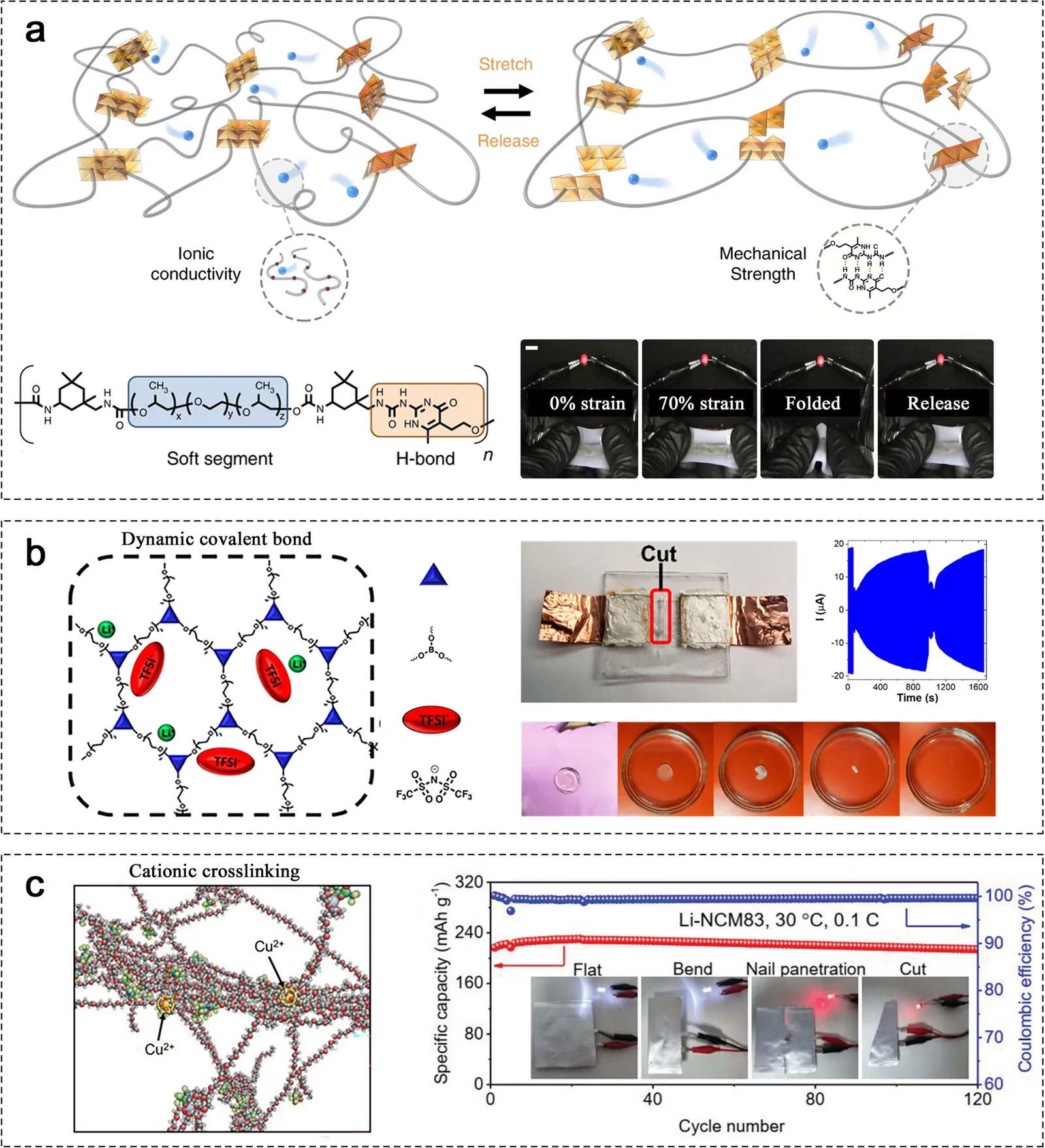
Schematic illustrations of physical or dynamic crosslinking strategies via hydrogen bonding, dynamic covalent bonding, and cation mediation. **a** Structure, operational principle, and tensile properties of a supramolecular Li⁺ conductor [135]. Copyright 2019, Springer Nature. **b** Synthesis of a self-healing and degradable SPEs based on dynamic physical crosslinking [137]. Copyright 2019, American Chemical Society. **c** 3D structure of a PEO/CuF_2_ electrolyte crosslinked by Cu^2^⁺ ions, along with its cycling performance and abuse tolerance [136]. Copyright 2023, John Wiley and Sons

3D networks constructed by dynamic crosslinking are ideal materials for flexible energy storage devices. Typically, Cui and Bao designed a series of supramolecular transiently crosslinked polymer electrolyte materials (Fig. 10a). Specifically, the PPO-*b*-PEO-*b*-PPO soft segments in this architecture serve as flexible lithium-conducting polymers with low *T*_g_ and high segmental mobility, and the multiple hydrogen bonds provided by the 2-ureido-4-pyrimidinone (UPy) groups create dynamic physical crosslinks between polymer chains. Therefore, it not only enhances toughness (~ 29 MPa) but also imparts excellent stretchability (~ 14 MPa) to the electrolyte, while maintaining high RT ionic conductivity of 1.2 × 10^−4^ S cm^−1^ [135]. The solid-state LMBs exhibited remarkable flexibility, maintaining excellent electrochemical performance under stretching and bending conditions. Concurrently, Evans et al. [137] developed a novel crosslinked network through dynamic covalent bonds between the hydroxy group of PEO and boric acid, achieving an ionic conductivity of 3.5 × 10^−4^ S cm^−1^ at RT, along with good self-healing and degradability properties (Fig. 10b). Notably, the dynamic networks SPEs can be readily dissolved in water to degrade to pure monomers and self-heal post-damage to recover inherent conductivity, demonstrating their application potential as sustainable solid electrolytes in flexible/wearable energy storage devices and other fields.

In 2023, our group incorporated a low content of CuF_2_ (< 0.5 wt%) into the PEO-based SPEs, utilizing the coordination of Cu^2+^ with both PEO segments and Li salt to construct a crosslinked structure (Fig. 10c) [136]. The Cu^2+^-coordinated PEO electrolyte significantly promotes the ionic transport in a molecular level through immobilizing TFSI^−^ anions and weakening interaction between Li^+^ and ether O of PEO chains, thereby achieving a high RT ionic conductivity of 2 × 10^−4^ S cm^−1^ and *t*_+_ of 0.42. Furthermore, this electrolyte membrane exhibits excellent mechanical strength with a tensile modulus of 57.3 MPa due to the crosslinking effect of Cu^2+^ in PEO matrix, which efficiently suppresses Li dendrites growth. Equipped with LiNi_0.83_Co_0.12_Mn_0.05_O_2_ cathodes, the pouch cells exhibit excellent capacity retention over 100 cycles at 30 °C (4.1 V cutoff) along with remarkable safety in abuse tests such as nail penetration, bending, and cutting.

#### Chemical Crosslinking

**Fig. 11:**
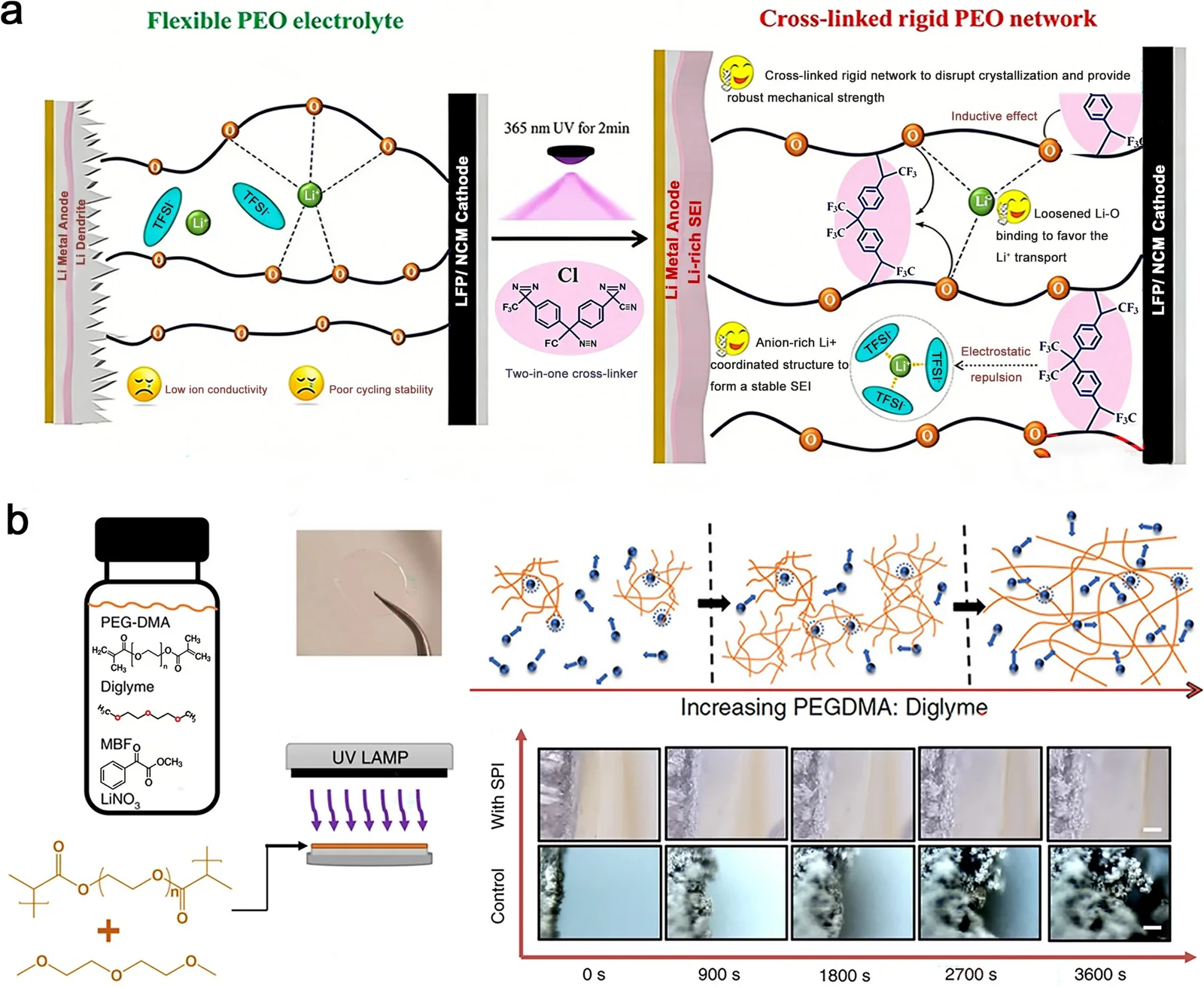
**a** Schematic illustration of in situ functional crosslinking for the rigid PEO electrolyte network [143]. Copyright 2025, John Wiley and Sons. **b** Synthesis strategy, architecture of the crosslinked PEGDMA network in the solid polymer interphase membrane, and the stability of corresponding Li electrodeposition morphologies [162]. Copyright 2019, Springer Nature

Compared to physical crosslinking, chemically crosslinking polymers exhibit superior geometric stability and good inhibition to Li dendrites growth, leading to broader applications in the field of PEO-based SPEs. Archer et al. [87] was the first to chemically crosslink polyethylene (PE) with PEO to obtain a crosslinked polymer electrolyte network. After incorporating an appropriate amount of liquid PEO molecules (*M*_n_ = 275 Da), this crosslinked electrolyte achieves a high RT ionic conductivity of 1.0 × 10^−4^ S cm^−1^, with a storage modulus of ~ 10^5^ Pa to effectively suppress Li dendrite growth—distinct from conventional BCPs dependent on high modulus theory. This result indicates that the robust nanoscale molecular network formed by chemical crosslinking can effectively provide excellent dendrite resistibility at the Li metal–electrolyte interface. In 2025, Ding et al. [143] developed a rigid polymer electrolyte network (PEO-*c*-C1) via in situ crosslinking 3,3′-((perfluoropropane-2,2-diyl)-bis(4,1-phenylene))bis(3-(trifluoromethyl)-3H-diazirine) (C1) and PEO (Fig. 11a). In this system, C1 functions as a structural crosslinker that enhances the tensile strength of the electrolyte to 0.595 MPa. Additionally, owing to the strong electron-withdrawing nature, it effectively weakened the Li–O coordination bonds, thereby promoting Li^+^ mobility and tailoring the anion-rich solvation structure to facilitate the formation of a stable LiF-rich SEI. As a result, PEO-C1 exhibited significantly enhanced ionic conductivity (1.4 × 10^−3^ S cm^−1^) and a high *t*_+_ (0.63) relative to those of PEO at 60 °C. The corresponding Li||LiFePO_4_ full cell demonstrated exceptional cycling stability with prolonged cycle life, highlighting the potential of this design for high-performance all-solid-state LMBs.

Chemical crosslinking can offer a robust and versatile platform to enhance the mechanical strength of all-solid-state PEO electrolytes while simultaneously providing a three-dimensional framework to immobilize liquid electrolytes in gel systems. Archer et al. utilized bifunctional polyethylene glycol dimethacrylate (PEGDMA) molecular chains as both cross linkers and polymerization monomers, directly crosslinking them in the ether solvent diglyme to create gel-type polymer solid electrolytes based on linear PEO networks via a facile ultraviolet (UV) light-initiated chemical reaction strategy (Fig. 11b) [162]. The systematic investigation into the effects of crosslinked network content on electrolyte performance revealed that at a crosslinked network content of 40 wt%, the GPEs exhibited a single-phase soft solid state. In this state, the oligomer ether demonstrated a sufficiently low ionic transport resistance, thereby achieving a high ionic conductivity of 1.0 × 10^−3^ S cm^−1^. Meanwhile, the dynamic interaction with the crosslinked network segments suppressed the overall convective motion, prevented unstable Li deposition caused by convection, and enhanced the electrochemical stability. Notably, in comparison to the uncoated control group, the Li surface coated with a solid polymer interface using the electrolyte exhibits a significantly smoother morphology during electrodeposition.

#### Inorganic Particle Cores

While conventional chemical crosslinking strategies have demonstrated effectiveness in suppressing Li dendrite growth, recent research has increasingly focused on inorganic particle-incorporated crosslinking strategies that can significantly enhance mechanical robustness and offer greater tunability in modular design, demonstrating great potential for developing high-performance PEO-based SPEs and practical LMBs at RT. Generally, inorganic particles are categorized into metallic and non-metallic types based on their chemical composition.

**Fig. 12:**
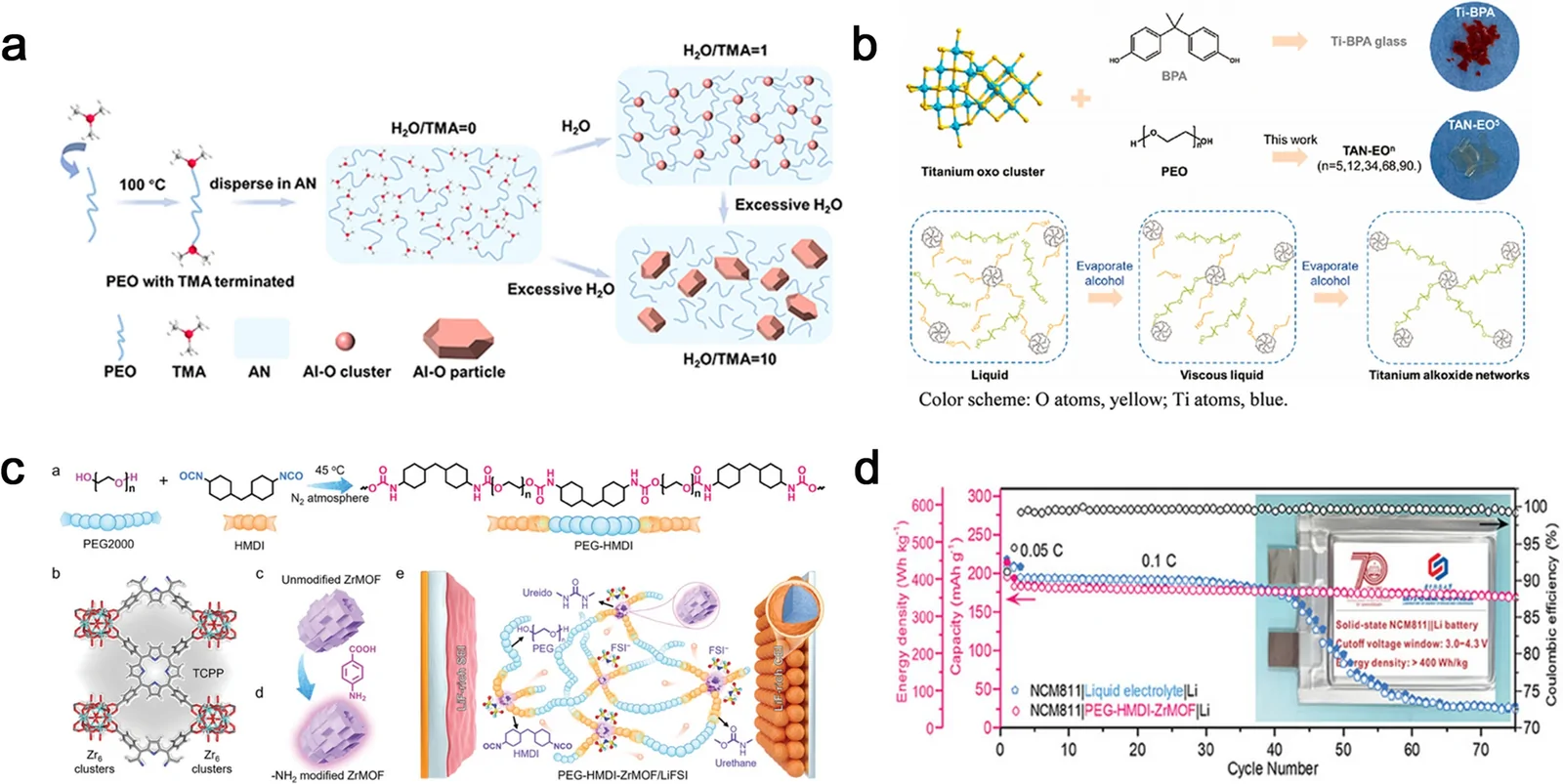
Chemical crosslinking strategies for metal-based inorganic particles with PEO. **a** Schematic illustration of the synthesis process of Al-O nanoclusters crosslinked PEO [163]. Copyright 2023, John Wiley and Sons. **b** Various ligands used in the synthesis of glassy MOFs and schematic of forming monolithic noncrystalline TANs [164]. Copyright 2023, American Chemical Society. **c** Schematic for the preparation of PEG-HMDI-ZrMOF and **d** cycling performance of Li|PEG-HMDI-ZrMOF| NCM811 pouch cells [144]. Copyright 2024, John Wiley and Sons

In 2023, Xie et al. [163] introduced a groundbreaking water-initiated crosslinking strategy in which the dynamic crosslinking of trimethylaluminum (TMA)-functionalized PEO chains are realized using water as an initiator with precisely controlled content through the in situ formation of ultrafine Al-O nanoclusters (Fig. 12a). This strategy enabled the creation of a three-dimensional interconnected network capable of incorporating ultrahigh plasticizer content (> 75 wt%) while maintaining exceptional mechanical properties, including a tensile strain of 4640% and toughness of 38.7 MPa. The dual-continuous phase structure not only provided robust mechanical support to suppress Li dendrite penetration but also established continuous ion-conducting channels, achieving a high ionic conductivity of 1.41 mS cm^−1^ at 30 °C. Consequently, Li||LFP batteries utilizing this electrolyte exhibited remarkable cycling stability with 98.6% capacity retention over 1000 cycles at 1 C, effectively reconciling the trade-off between ionic conductivity and mechanical integrity in SPEs.

Building upon this innovation, the researchers further exploited the modular chemistry of metal–organic frameworks (MOFs) to design polymer network solid electrolytes. In this strategy, PEO chains replaced conventional short-chain ligands as organic linkers, coordinating with nanoscale titanium oxide clusters to form titanium alcohol networks (TANs) (Fig. 12b) [164]. The TAN platform allows precise tuning of two key parameters: (1) molecular weight of PEO linkers to optimize chain flexibility (enhancing ionic mobility) and (2) crosslinking density of the coordination network to regulate mechanical strength. Batteries employing TAN-EO/Li electrolytes exhibited high electrochemical stability, retaining 92.4% capacity (128.7 mAh g^−1^) after 500 cycles at 0.5 C. This modular design paradigm establishes a universal framework for balancing ion transport and mechanical resilience in solid-state battery systems.

Compared with traditional inorganic particle, which often exhibit non-uniform particle sizes and a high tendency for agglomeration, zirconium oxide-based MOFs have emerged as one of the most promising classes of functional nanomaterials due to their well-defined nanostructure and tunable surface chemistry. Recently, our group developed a novel SPEs through covalent integration of amino-functionalized zirconium porphyrin-based MOFs (ZrMOFs) with short-chain PEG [144]. In this design, ZrMOFs act as special multi-point crosslinkers and chain extenders, reacting with the end groups of PEG-HMDI to obtain a crosslinking interpenetrating SPEs with a bridging structure (PEG-HMDI-ZrMOF) (Fig. 12c). The electrolyte exhibits outstanding mechanical strength (76.5 MPa) and excellent toughness (≈ 2050%). Furthermore, the abundant ether and carbonyl oxygen atoms, along with accessible Lewis acid sites, facilitate a high ionic conductivity of 5.7 × 10^4^ S cm^−1^ at 30 °C and a *t*_+_ of 0.84. Notably, a 1.5-Ah pouch cell delivered an impressive energy density of 446 Wh kg^−1^ and retained 90.7% of its initial capacity after 220 cycles (Fig. 12d), demonstrating the potential of this electrolyte for practical applications in high-energy–density solid-state LMBs.

**Fig. 13:**
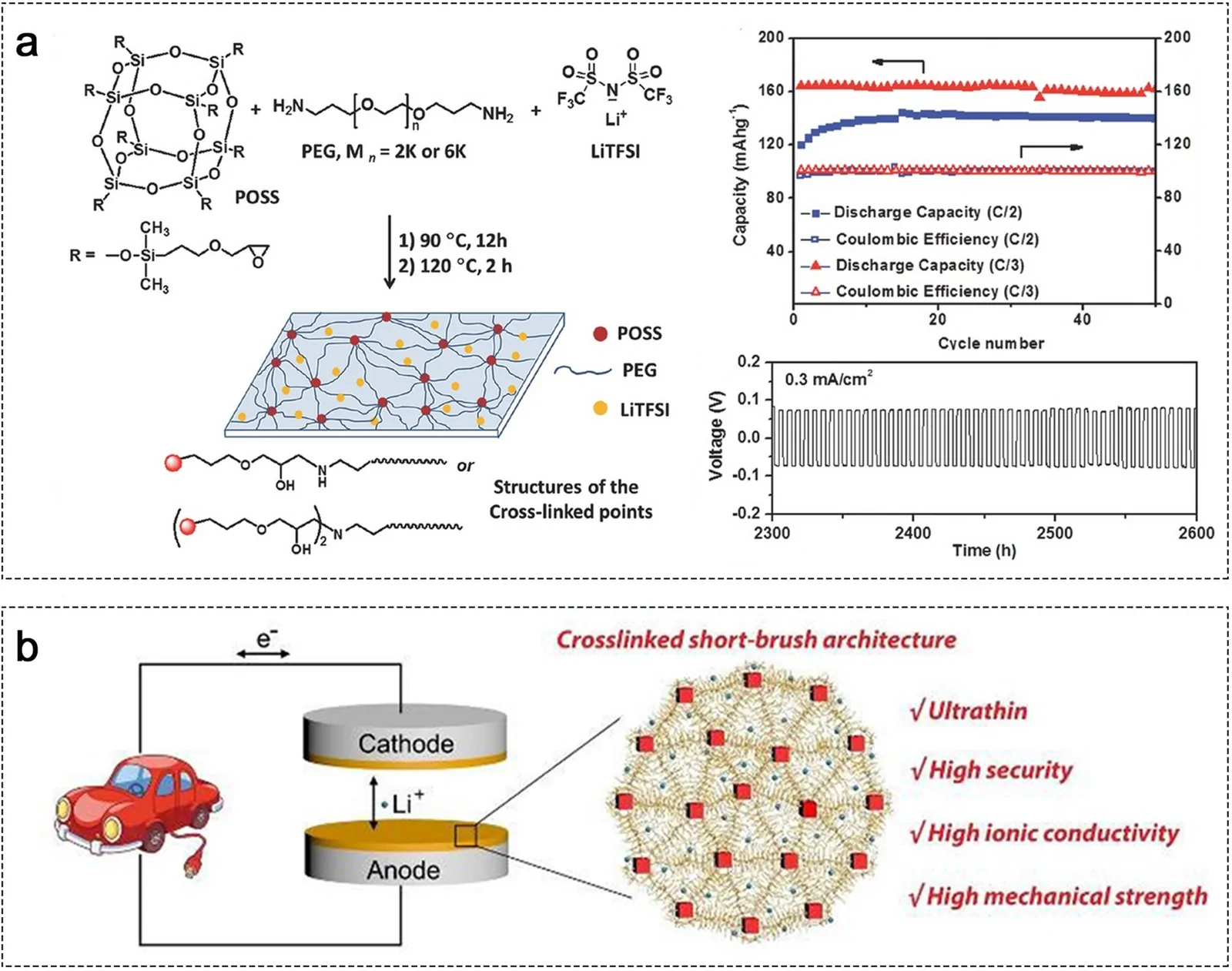
Chemical crosslinking strategies for non-metallic inorganic particles with PEO. **a** Schematic of POSS crosslinked networks with linear PEO backbone, and its corresponding cycling performance [166]. Copyright 2015, John Wiley and Sons. **b** Schematic of POSS crosslinked structure with short-brush PEO side chain enabling high mechanical strength and conductivity at RT [139]

In addition to metal-based inorganic particles, non-metallic inorganic particles can also serve as chemical crosslinking cores to enhance the mechanical strength and electrochemical performance of SPEs. Archer et al. fabricated a similar PEO crosslinked network structure using SiO_2_ microspheres as the crosslinking core. This structure serves as a polymer matrix to absorb propylene carbonate solvent, forming a GPEs with a RT ionic conductivity of 5.0 × 10^−3^ S cm^−1^ and a storage modulus of 10^6^ Pa, thereby enabling stable operation of the Li||LFP quasi-solid-state battery at RT [165]. Pan et al. [166, 167] developed an inorganic–organic crosslinked network using POSS as the crosslinking core and PEO molecular chains as Li^+^-conducting media (Fig. 13a). By tailoring the degree of crosslinking and the length of the PEO chains, this inorganic–organic network achieved a RT ionic conductivity ranging from 10^−5^ to 10^−4^ S cm^−1^, and a Li symmetric cell could stably operate for 2600 h at 90 °C, exhibiting excellent dendrite resistance, while the Li||LFP all-solid-state LMBs demonstrated outstanding cycling stability at 90 °C.

However, both types of crosslinked network solid electrolytes suffer from limited segmental mobility of the PEO chains, which still necessitates the introduction of high temperatures or organic solvents to achieve excellent battery performance. To address this, our group combined the crosslinked network with a grafted brush-like structure, designing a PEO brush network structure. Through an in situ photoinitiated film-forming process, we produced a solid electrolyte film that integrates high ionic conductivity, excellent dendrite resistance, high thermal stability, and fire resistance (Fig. 13b) [139]. Compared to the crosslinked linear network, the short PEO chains released at one end of the crosslinked brush network possess more free volume, allowing for enhanced segmental mobility through molecular structure modulation. This innovative approach elevated the RT ionic conductivity of PEO-based all-solid-state electrolytes to the order of 10^−4^ S cm^−1^ for the first time. Additionally, the rigid POSS particles served as reinforcing phases, constructing a three-dimensional network structure that endowed the electrolyte film with exceptional mechanical strength (57 MPa) and thermal stability (≥ 300 °C). The resulting all-solid-state LMBs demonstrated stable cycling for over 50 cycles at RT.

In summary, three-dimensional molecular network constructed by dynamic or permanent crosslinking offers a great superiority in mechanical strength and dendrite growth suppression, a simple synthetic route, and a strong feasibility for scale-up thin-film preparation, making them one of the most active research hotspots in the past 5 years. Accordingly, LMBs utilizing these strategies exhibit excellent performance. Specifically, dynamic crosslinking demonstrates obvious mechanical toughness (stretching and bending endurance) and self-healing ability, which is significant to meet the requirements associated with wearable electronic devices and intelligent robots. Chemical crosslinking, achieved through soft segments, rigid segments, or inorganic particles, forms tightly interconnected networks that confer superior geometric and electrochemical stability. This approach provides robust mechanical strength and stronger Li dendrite suppression ability, with performance being precisely tunable by regulating the type and degree of crosslinking agents. However, it should be noted that crosslinked network tends to restrict the segmental relaxation of PEO chains, necessitating elevated temperature or plasticizer addition to enhance ionic conductivity. Truly achieving RT-available all-solid-state LMBs requires persistent efforts. Our proposal of crosslinked short-brush architecture provides a promising research direction, with emphasis on rational integration of advantages in typical molecular engineering strategies.

## Chemical Design

### Anion-Anchored Design for High ***t***_+_

In conventional SPEs, the polymer matrix dissociated Li salt into Li^+^ and anions,which both participate in ion transport, but the latter is invalid for cell charge-transfer process. Although a high dielectric constant of polymer matrix is need, such as PEO, to dissolve Li salt, it also leads to a strong coordination with Li^+^, resulting in a *t*_+_ much lower than 0.5 (≤ 0.2 for PEO system) [168]. Moreover, according to space-charge theory the depletion of anions at the electrode surface would produce space-charge field, which is considered to be a main culprit for Li dendrite growth [169]. In Li/polymer cells, Sand’s time (*τ*) is generally utilized to describe the onset of Li dendrite growth, as shown in Eq. (2):

2 $$ \tau = \pi D\left( {C_{0} e/2Jt_{a} } \right)^{2} \quad {\mathrm{with}}\;t_{a} = \mu_{a} /\mu_{a} + \mu_{c} $$

where *J* denotes the current density, *D* represents the diffusion coefficient, *μ*_*a*_ and *μ*_*c*_ are the anion and cation transference numbers, respectively, e is the elementary charge, and *C*_0_ refers to the initial cation concentration. At a constant *J*, increasing *t*_+_ or suppressing anion migration in electrolytes is a critical approach to delay the onset of dendrite growth, thus effectively suppressing Li dendrite formation. In this context, anion-anchored engineering, as a core chemical design strategy, essentially alters the chemical composition and coordination environment of the electrolyte by covalently anchors anion groups onto the polymer backbone, enabling the exclusive long-distance migration of Li^+^. This approach has emerged as an effective solution and thus been intensely developed [170–173].

#### Polyanion Design

**Fig. 14:**
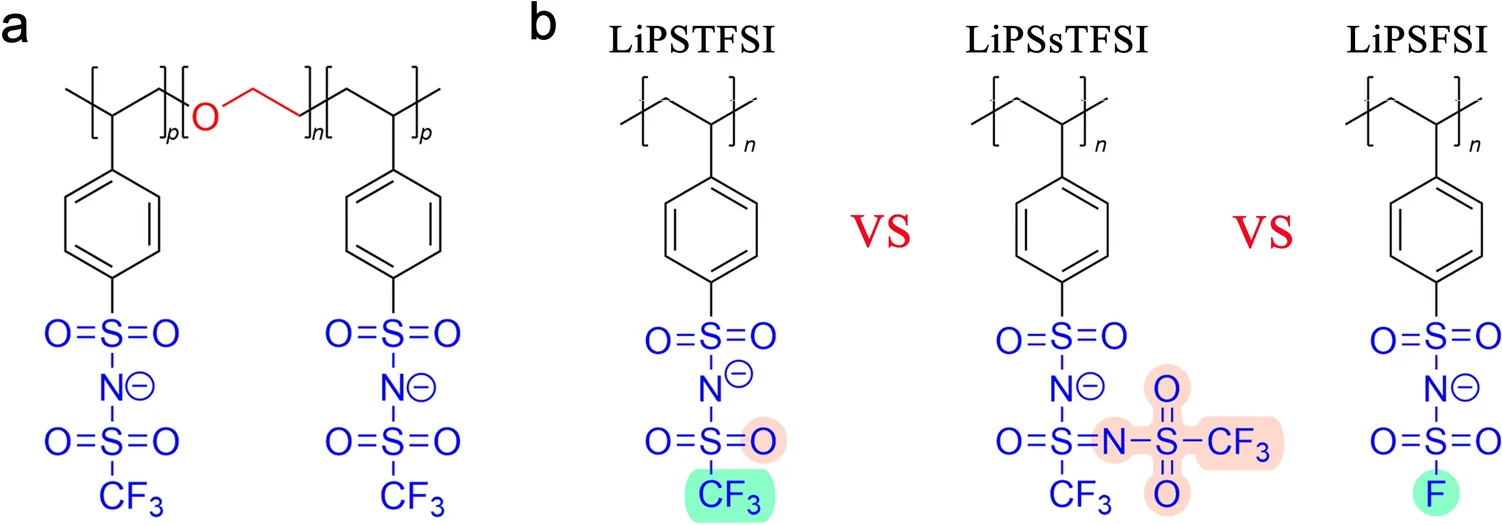
**a** Molecular structure of typical PEO-type SIPEs. **b** Chemical structures of the different anion group-centered structures (LiPSTFSI, LiPSsTFSI, LiPSFSI)

Armand et al. [174] initiated the earliest anion-anchored strategy into PEO-based SPEs by synthesizing ionic polymers, such as lithium poly(4-styrenesulfonyl (trifluoromethanesulfonyl)imide) (LiPSTFSI), lithium poly(styrene sulfonate) (PSLi), and applying them to blend with PEO. As expected, the *t*_+_ approaches 1 and the conductivity increases by one order of magnitude than that of PSLi system. On this basis, they change the physically blending strategy into chemical polymerization, creating the first PEO-based single-ion polymer electrolytes (SIPEs) as shown in Fig. 14a [170]. This anion-anchored PEO-based SPE inherits the high mechanical property of PEO-*b*-PSt block copolymers and exhibits a desirable *t*_+_ higher than 0.85. Moreover, since the anion groups are fixed on polymer backbones, negative charges are lost (oxidation side reaction) at the interface only, enlarging the ESW of PEO-based SPE up to 5 V (vs. Li/Li^+^).

Charge density of anion groups is a key factor that determines the ionic dissociation and transport. In the past years, while numerous anionic centers, such as carboxylates (–COO^−^) [175, 176], sulfonate (–SO_3−_) [177, 178], and sulfonimide anion (–SO_2_N^(−)^SO_2−_) [170, 174, 179, 180], have been extensively investigated, –SO_2_N^(−)^SO_2−_ are still the frontrunner and most widely used due to their more delocalized negative charge structure and reduced charge density. Specifically, Zhou et al. [181, 182] systematically investigated the structural effects of negatively delocalized anionic groups in LiPSTFSI. Two modified anion structures were designed by replacing the =O group with the strong electron-withdrawing =NSO_2_CF_3_ group and substituting –F for –CF_3_ within the –SO_2_N^−^SO_2_CF_3_ (TFSI^−^), yielding –SO_2_N^−^–SO (= NSO_2_ CF_3_) CF_3_ (sTFSI⁻) and SO_2_N⁻–SO_2_F (FSI⁻), respectively. Corresponding ionic polymers of LiPSsTFSI and LiPSFSI were synthesized as shown in Fig. 14b. Compared to the LiPSTFSI/PEO system, the LiPSsTFSI/PEO electrolyte exhibited enhanced anionic charge delocalization and improved ionic conductivity, while the LiPSFSI/PEO system demonstrated superior compatibility with Li metal electrode via probably generating stable intermediate phases.

#### Topology Optimization

**Fig. 15:**
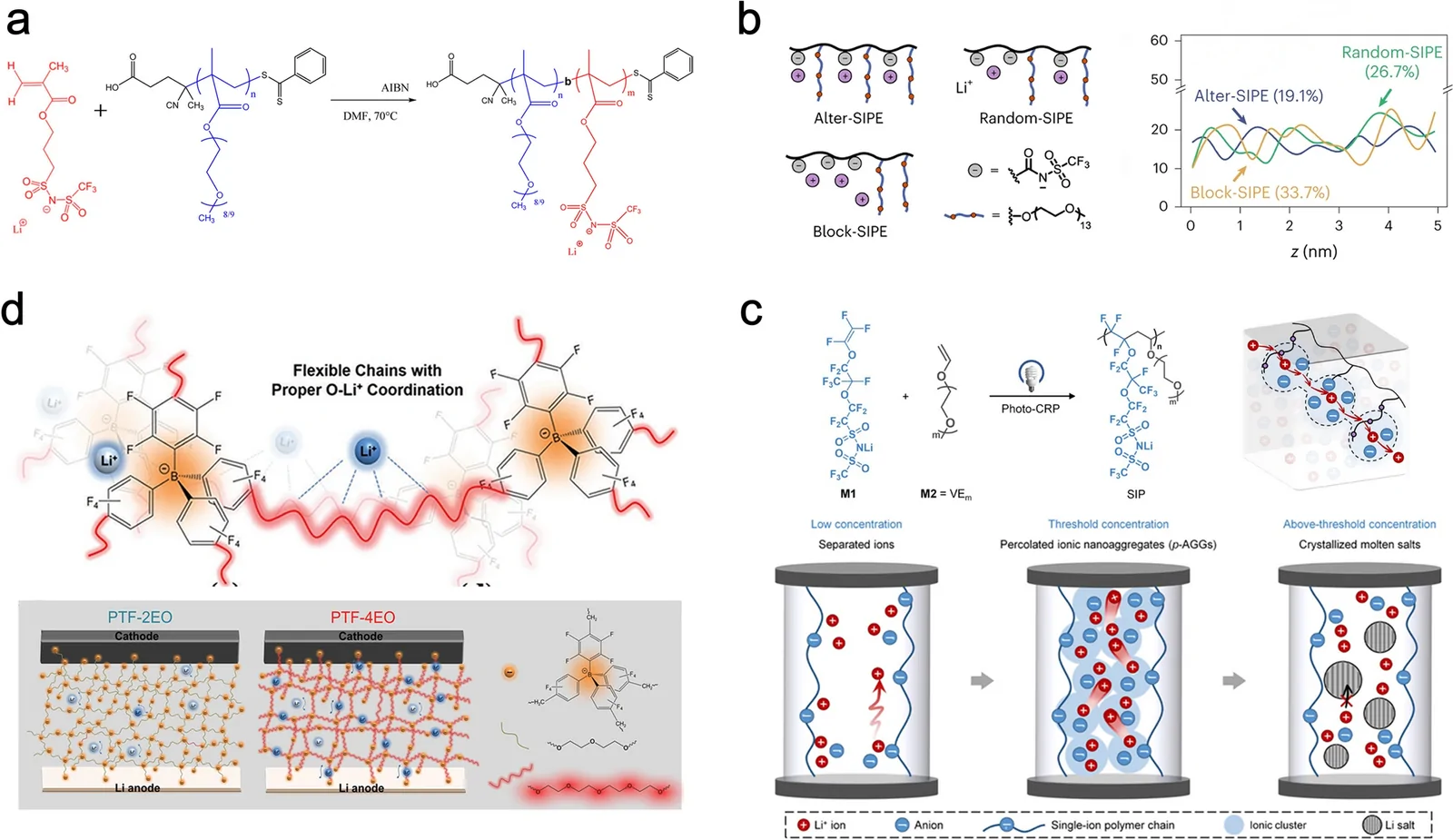
SIPEs with topological optimization compensating for ionic conductivity or mechanical strength. **a** Structure and synthesis of poly(PEGM)-*b*-poly(LiMTFSI) copolymer as a brush-like SIPE [180]. Copyright 2016, American Chemical Society. **b** Brush-like SIPE with alternating-sequence architecture and its comparison of Li^+^ density distribution standard deviation to traditional random or block copolymers [185]. Copyright 2023, Springer Nature. **c** Synthesis and ion distribution mechanisms of alternating-sequence brush-like SIPE as a function of Li salt concentration, with emphasis on an ideal polymer-in-salt pattern [173]. Copyright 2025, American Chemical Society. **d** Organic borate group functions not only as an anion centers, but also as a cross linker for flexible PEO segments to enhance rigidity and dendrites resistance of SIPEs [186]. Copyright 2022, John Wiley and Sons

Ionic conductivity of anion-anchored SPEs is primarily governed by the extent and connectivity of amorphous phase within PEO chains. Generally, these materials demonstrate ionic conductivity at 10^−5^ S cm^−1^ level at 60 °C. Below 60 °C, increased crystallinity of PEO restricts the segmental motion, resulting in an extremely low ionic conductivity, typically of 10^−9^~10^−7^ S cm^−1^ at RT [183, 184]. Therefore, translating the linear long PEO chains into short side chains to construct brush topologies is a sought-after pathway to explore high-performance anion-anchored electrolytes. For instance, Gerbaldi et al. [180] reported a series of brush-like diblock copolymer SIPEs based on anion-anchored poly(LiMTFSI) and poly(PEGM) (Fig. 15a). The optimized electrolyte with 8 EO units in its side chains displays a low *T*_g_ (down to − 61 °C) and an amorphous liquid-like characteristic, achieving a RT ionic conductivity as high as 2.3 × 10^−6^ S cm^−1^ (*t*_+_ approaching 1).

In 2023, Chen et al. [185] further created an alternating-sequence SIPE (Alter-SIPE) through photo-controlled radical alternating copolymerization (Photo-CRAP), in which each anion-anchored polymer unit periodically paired with a PEO side chain (Fig. 15b). Compared to conventional or randomly distributed brush-like block SIPEs, molecular dynamic simulation reveals that the periodically alternating architecture exhibits a lower standard deviation coefficient of the average Li^+^ density, indicating a more uniform Li^+^ distribution and reduced local Li^+^ aggregation. As a result, the enhanced dissociation and uniform distribution of Li⁺ grant the Alter-SIPE a markedly improved ionic conductivity up to 4.2 × 10^−5^ S cm^−1^ at RT, without any addition of plasticizers. The constructed PEO-based all-solid-state LMBs exhibited stable cycling performance over 40 cycles, marking a significant advancement in the practical application of all-solid-state PEO-based electrolytes at ambient conditions. More recently, the same group further reported a periodic sequence-controlled single-ion polymer-in-salt (SIP-in-salt) electrolyte, synthesized from lithium perfluorosulfonylimide vinyl ether (M1) and PEO-substituted vinyl ether (M2) via photo-CRP (Fig. 15c) [173]. Upon reaching the threshold salt concentration, this SSE not only enables uniform distribution of abundant Li^+^ under high-salt conditions but also undergoes a structural transition from predominant contact ion pairs (CIPs) to the formation of percolating ionic nanoaggregates (*p*-AGGs). The authors propose that the *p*-AGGs in the SIP-in-salt may form an “interconnected” path, where the addition of salt shortens the hopping distance of Li^+^ in *p*-AGG, and the TFSI^–^ acts as “springboards” to promote the hopping of Li^+^ in a more continuous and efficient manner. This electrolyte exhibits high ionic conductivity (3.9 × 10^−5^ S cm^−1^) with *t*_+_  = 0.85 at 25 °C.

High ionic conductivity in SIPEs is typically accompanied by a trade-off in mechanical properties. To ensure adequate mechanical performance, a substantial electrolyte membrane thickness is often required, which unfortunately increases the overall cell resistance and compromises energy density. The molecular design of a crosslinked network featuring anions as linker enables simultaneous improvement in both the mechanical strength and ionic conductivity of SIPEs. Lian et al. [186] developed an interpenetrating single-ion network polymer via the crosslinking of lithium tetra(4-chloromethyl)-2,3,5,6-tetrafluorophenylborate with four EO units (PTF-4EO) (Fig. 15d). This innovative structure enables PTF-4EO to exhibit weakly interacting anions and the unique diamondoid network with coordinating ether oxygen segments, thereby achieving superior electrochemical performance. Specifically, PTF-4EO with the plasticizer PC demonstrates a RT ionic conductivity of up to 3.53 × 10^−4^ S cm^−1^, a *t*_+_ as high as 0.92, an ESW exceeding 4.8 V (vs. Li^+^/Li), and excellent mechanical strength with a modulus of 1.3 GPa.

In summary, to overcome the low *t*_+_ and dendrite growth issues of traditional PEO-based SPEs, significant progress has been made in SIPEs through ingenious design of polyanions and further topological optimization. Typical data are summarized in Table 3. The design of polyanions, which covalently anchor anions to the polymer backbone, not only significantly enhances ionic conductivity with a *t*_+_ approaching one but also broadens the ESW. Meanwhile, topological structure optimizations such as random and alternating-sequence brush-like structures as well as crosslinked network structures effectively suppress PEO crystallization, optimize the Li^+^ distribution path, and synergistically enhance mechanical strength, thereby increasing the RT ionic conductivity to the 10^−5^–10^−4^ S cm^−1^ range. This has strongly promoted the practical application of PEO-based all-solid-state batteries.

**Table 3:** Performance comparison of PEO electrolytes based on chemical design

Entry	Polymer matrix	Ionic conductivity (S cm^−1^)	*E*_ox_ (V vs. Li/Li^+^)	*t*_+_	Cycling performance	Electrode materials	References
*Anion-anchored*							
1	P(STFSILi)-*b*-PEO-*b*-P(STFSILi)^a)^	1.3 × 10^−5^ (60 °C)	5.0	> 0.5	C/15 to 2 C; 90th (60 ~ 80 °C); Solid	Li||LFP	[170]
2	Alternating-sequence SIPE	4.2 × 10^−5^ (30 °C)	–	0.93	0.066 mA cm^−2^; 40th (30 °C); Solid	Li||LFP	[185]
3	Fluoroalkyl-functionalized alternating-sequence SIPE	3.9 × 10^−5^ (RT)	5.0	0.85	0.1 C; 40th (25 °C); Solid	Li||LFP	[173]
4	BC-*g*PLiSTFSI-*b*-PEGM^b)^	1.3 × 10^−5^ (30 °C)	–	> 0.85	1 C; 300th (30 °C); Quasi-solid	Li||LFP	[187]
5	LiCTFPB-*c*-TEG^c)^	3.53 × 10^−4^ (RT)	4.8	092	0.5 C; 200th (30 °C); Solid	Li||NCM712	[186]
6	Poly(PEGM)-*b*-poly(LiMTFSI)^d)^	2.3 × 10^−6^ (RT)	4.5	0.83	0.1 C; 100th (70 °C); Solid	Li||LFP	[180]
*Backbone modification*							
7	PEG/PFPE^e)^	2.26 × 10^−5^ (60 °C)	6.0	–	0.1 mA cm^−2^; 200 h (80 °C); Solid	Li||Li	[188]
8	PEO-Mg–Al-LiTFSI^f)^	2..3 × 10^−4^ (RT)	> 5	0.67	0.1 C; 100th (60 °C); Solid	Li||Li-rich Li_1.14_Ni_0.136_Co_0.136_Mn_0.542_O_2_	[189]
9	PEO-*g*FN^g)^	1.01 × 10^−4^ (RT)	0.47↑	0.51	1 C; 100th (25 °C); Solid	Li||LFP	[190]
*Terminal modification*							
10	PEGDME^h)^	∼ 1.5 × 10^−4^ (60 °C)	4.3	–	0.2 C; 100th (60 °C); Solid	Li||NCM532	[191]
11	PEO/TEGDMA/TEGDME^i)^	2.7 × 10^−4^ (24 °C)	5.38	0.56	0.1 C; 100th (RT); Solid	Li||LFP	[192]
12	*α*-CD-PEO-*g*PCL^j)^	1.0 × 10^−4^ (RT)	4.7	0.6	1 C; 200th (60 °C); Solid	Li||LFP	[193]
13	MPEG-3F^k)^	8.71 × 10^−4^ (RT)	4.65	0.63	0.2 C; 300th (RT); Liquid	Li||LFP	[194]

a) *P(STFSILi)* poly(styrene trifluoromethanesulfonylimide of lithium); b) *BC* bacterial cellulose, PLiSTFSI = poly(lithium 4-styrenesulfonyl-(trifluoromethylsulfonyl) imide), *PEGM* poly(diethylene glycol monomethyl ether methacrylate); c) *LiCTFPB* lithium tetrakis(4-(chloromethyl)-2,3,5,6-tetrafluorophenyl)borate salt, TEG = tetraethylene glycol; d) *poly(PEGM)* poly(ethylene glycol) methyl ether methacrylate, poly(LiMTFSI) = poly(lithium 1-[3-(methacryloyloxy)propylsulfonyl]-1-(trifluoromethylsulfonyl)imide); e) *PFPE* perfluoropolyethers; f) the Mg^2+^ and Al^3+^-coordinated PEO-based electrolyte; g) *FN* fumaronitrile; h) *PEGDME* polyethylene glycol dimethyl ether; i) *TEGDMA* triethylene glycol dimethacrylate; *TEGDME* triethylene glycol dimethyl ether; j) *CD* cyclic cyclodextrin; *PCL* caprolactone; k) *MPEG-3F* trifluoroethoxy-terminated methoxy polyethylene glycol

### HOMO Energy Regulation for Extended ESW

**Fig. 16:**
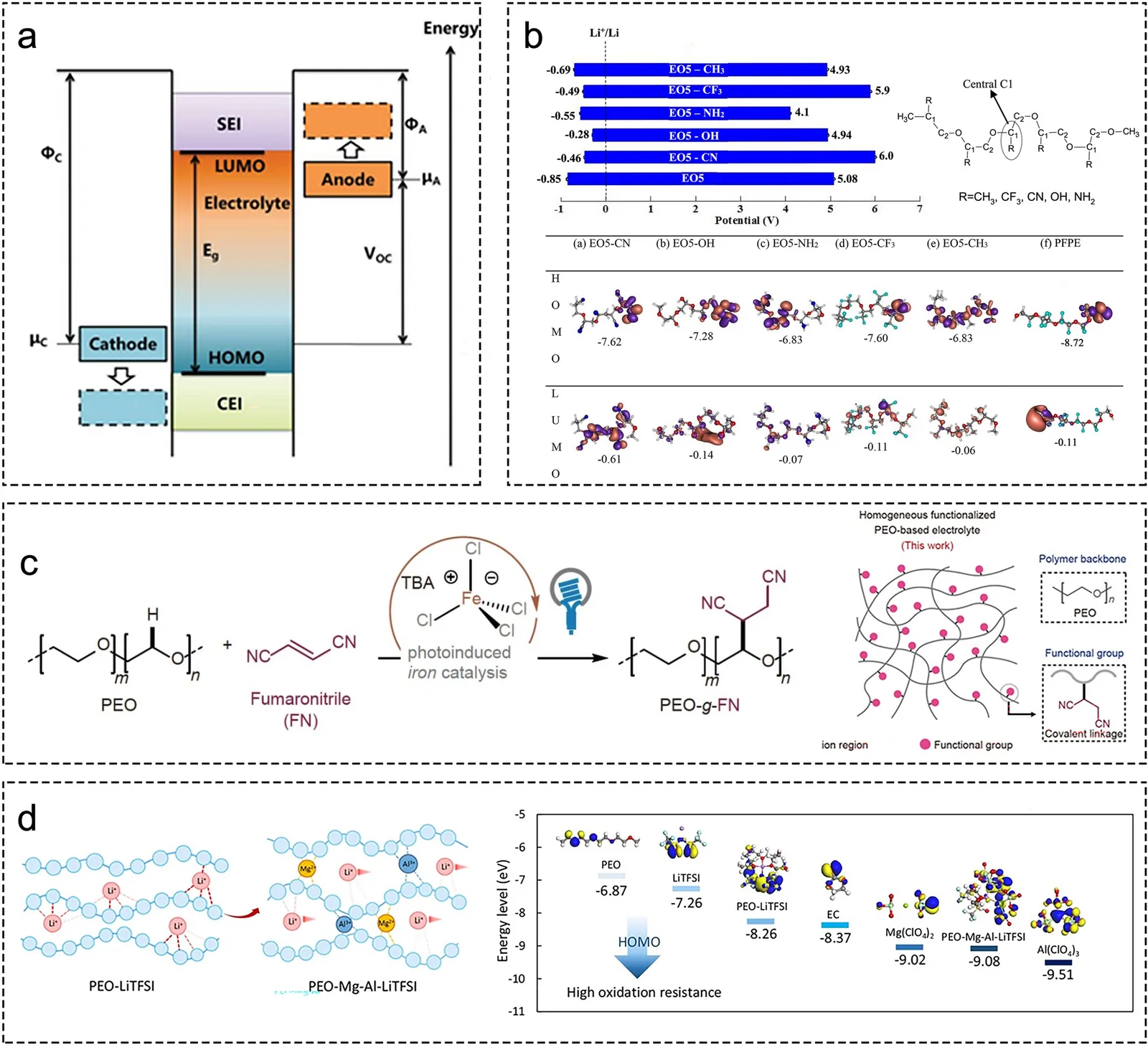
**a** Schematic energy diagram of the SPEs. Eg is the energy gap between the HOMO and LUMO of the SPEs [69]. Copyright 2019, John Wiley and Sons. **b**–**d** Backbone modification strategies for PEO: **b** effect and schematic diagram of the redox potential of various functional groups substituted on PEO(EO5) electrolyte and HOMO/LUMO orbitals [203]. Copyright 2018, Elsevier. **c** Synthesis and schematic of SN-functionalized PEO-based electrolyte [190]. Copyright 2024, Science Press. Copyright. **d** Schematic of the function of Lewis acid additives in PEO-LiTFSI and PEO-Mg-Al-LiTFSI electrolytes and the HOMO energy levels of each component. Copyright 2024, Springer Nature

As previously discussed, significant progress has been made in the macromolecular engineering design of PEO-based SPEs with respect to ionic conductivity, *t*_+_, and mechanical strength. However, the limited ESW (< 4 V) [195] of PEO significantly restricts their compatibility with high-voltage cathode materials for achieving solid-state batteries with higher energy density. As illustrated in Fig. 16a, designing high-voltage SPEs should be approached by reducing the HOMO energy level of the polymer matrix so that the cathode potential (*μ*_c_) exceeds the HOMO energy of electrolytes; alternatively, a chemically passivated intermediate phase, namely the cathode–electrolyte interphase (CEI), that can be formed on the cathode material surface [69]. During the Li intercalation and deintercalation process, the volume expansion of cathode material often induces cracking in the CEI, re-exposing the active material, and triggering side reactions with PEO—specifically, due to the low onset decomposition voltage of PEO-based electrolytes and the surface catalytic effect of highly oxidized Ni^4+^ (Co^4+^ or Mn^4+^), PEO undergoes electrochemical oxidation to generate products such as C_2_H_2_, C_2_H_4_, CO_2_, CO, and C_2_H_6_ [196, 197]. These products will simultaneously hinder the transport of lithium ions in the SPE; additionally, hydrated bis(trifluoromethanesulfonyl)imide anions (HTFSI) undergo transmembrane migration to the anode side, where they react with lithium metal to produce H_2_ [198]. Furthermore, the chemical redox reaction occurring between the highly reactive cathode and PEO-based SPEs will lead to the structural collapse of the cathode [199]. Such behaviors are considered the main cause of rapid capacity fading, increased internal resistance, and reduced thermal safety. To fundamentally address the poor compatibility between PEO and high-voltage cathode materials, researchers have explored lowering the HOMO energy level of PEO and enhancing its oxidation resistance via chemical modification strategies at the molecular level of PEO, including terminal-OH modification [190, 191, 194, 200–202], EO backbones modification [188–190, 203], and some other strategies [204–207].

#### Backbone Modification

##### Electron-Withdrawing Group Substitution

The ether group exhibits relatively low oxidation stability, which unfortunately is incompatible with high-voltage-operating cathodes such as NCM and LCO [20, 25, 208]. Lee et al. [203] investigated the ESW of polyether-based SPEs with different functional groups substituted on the PEO backbone through density functional calculations. The simulations demonstrated that for isolated EO5 and Li salt systems, the *E*_ox_ increased to approximately 6 V (vs. Li^+^/Li) upon functionalizing ethylene oxide (EO) with electron-withdrawing groups such as cyano (–CN) and –CF_3_. This is attributed to the reduced electron cloud density of backbone induced by –C–F and –C≡N functional groups, which significantly suppress the likelihood of further electron removal via oxidation, thereby enhancing the oxidation stability. This is reflected in HOMO energy level of EO5-CN (− 7.6 eV) and EO5-CF_3_ (− 7.6 eV), which are 0.8 V lower than those of PEO (Fig. 16b). Additionally, perfluoropolyether (PFPE), as fully fluorinated analogs of PEO, is also calculated to have a lower energy level of the lowest unoccupied molecular orbital (LUMO) (corresponding to reduction potential) and a higher HOMO in an isolated state, thereby enhancing the redox stability of the polymer salt system and expanding the ESW (> 1 V).

Although electrolytes like PFPE can enhance oxidation stability through fluorination, they suffer from insufficient Li^+^ coordination and poor ion transport. To address this, Amanchukwu et al. [188] introduced crosslinking polyethylene glycol (PEG) units into the PFPE matrix. Blending PEG and PFPE in varying proportions induces microscale phase separation, while the weak ions–PFPE interaction promotes salt phase separation in the PEG region and facilitates ion transport therein. This design enhances the electrolyte conductivity by six orders of magnitude, from 1.55 × 10^−11^ to 2.26 × 10^−5^ S cm^−1^ at 60 °C. Moreover, this electrolyte exhibits a high *E*_ox_ above 6 V (vs. Li^+^/Li) compared to 4.6 V (vs. Li^+^/Li) for pure crosslinking PEG. However, how the strong electronegativity of the F atoms in the PFPE system affects the charge distribution of the polyether segments and regulates ion migration remains a key scientific issue that needs to be urgently addressed.

The Li^+^ transport mechanism in PEO-based electrolytes primarily involves slow ion hopping between adjacent coordination sites. Conventional strategies, such as incorporating SN as a plasticizer, can effectively optimize both the spatial environment (free volume) and the chemical environment (coordination structure) to facilitate ion transport. However, the use of large quantities of low-*M*_n_ solvents introduces potential safety risks, including volatility and flammability [209]. Ding et al. [190] employed a direct C–H functionalization approach by grafting fumaronitrile (FN) into the PEO backbone, thereby synthesizing a uniformly SN-functionalized PEO-based electrolyte (PEO-*g*FN), which integrates the low HOMO energy level and coordination properties of SN directly into the polymer framework (Fig. 16c). Consequently, the SN functional group enhances the conformational disorder and segmental mobility of PEO chain while serving as an effective site for rapid interchain Li^+^ conduction, thus enabling a high ionic conductivity of 1.01 × 10^−4^ S cm^−1^ at 25 °C. Meanwhile, the strong electron-withdrawing –CN in the functional moiety partially suppresses electrochemical oxidation of the PEO backbone, thus leading to an expanded ESW for the SPEs.

##### Coordination Effect of Lewis Acid

Recently, Wang et al. [189] reported a Lewis acid-coordinated PEO-based electrolyte designed for 4.8 V-class high-energy batteries. The incorporated Mg^2+^ and Al^3+^ ions exhibit strong electron-withdrawing capabilities, effectively reducing the electron density of the ether oxygen (EO) chains via chelation within the coordination structure (Fig. 16d). This mechanism mitigates the reactivity between the EO groups and the LiNi_0.83_Co_0.12_Mn_0.05_O_2_ cathode, thereby significantly enhancing the interfacial compatibility of the battery at 4.8 V. Furthermore, the characteristic coordination structures formed by Mg^2^⁺, Al^3^⁺, and additional anions exhibit a 2.21 eV reduction in HOMO energy while promoting the formation of inorganic-rich interfacial phases compared to pristine PEO (Fig. 16e). Consequently, the Mg^2+^ and Al^3+^-coordinated PEO-based electrolyte (PEO-Mg-Al-LiTFSI) exhibits both a high *E*_ox_ (exceeding 5 V) and favorable ionic conductivity (0.23 mS cm^−1^ at RT). Utilizing this optimized electrolyte enables a stable performance over 300 cycles in all-solid-state LMBs.

#### Terminal Modification

In macromolecular engineering, the overall oxidation resistance of the polymer matrix can be significantly enhanced by constructing more stable macromolecular structures through the substitution of PEO end groups. Generally, terminal hydroxyl groups (–OH) can be replaced with relatively more oxidation-resistant ether groups (such as methoxy groups or trifluoroethoxy groups). Moreover, the introduction of stable terminal ester groups can also effectively suppress the reactive interaction between ether groups at the terminal or side chains and the positive electrode.

##### Ether Group

**Fig. 17:**
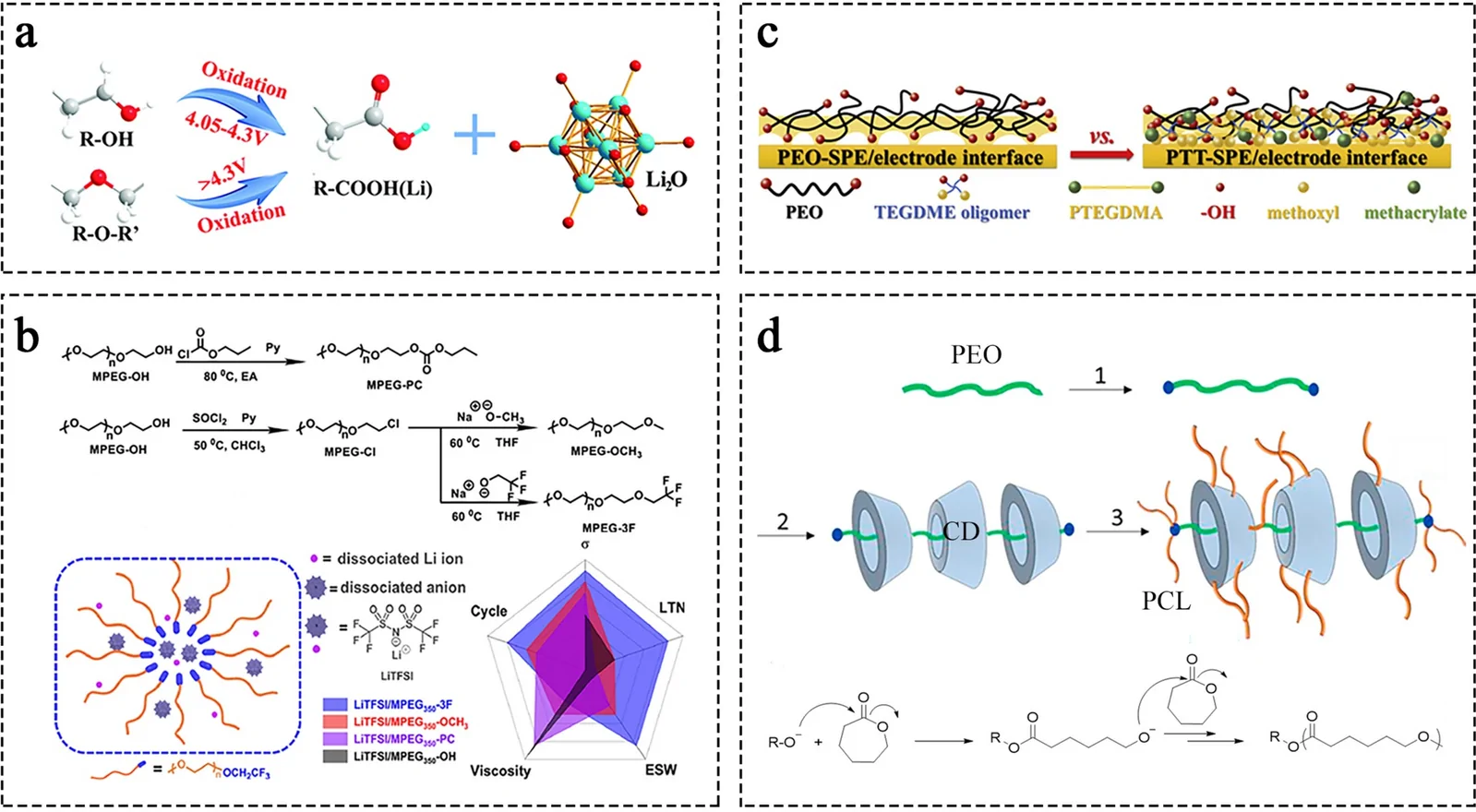
Terminal modification strategies for PEO via ether or ester functional groups. **a** Possible oxidation mechanism of ether-based polymers at high voltages [191]. Copyright 2020, Royal Society of Chemistry. **b** Physical and electrochemical properties of four kinds of PEG-based electrolytes with different terminals and formation of the large-sized aggregates in LiTFSI/MPEG-3F [194]. Copyright 2023, Elsevier. **c** Schematic representation of the PEO/PPT-SPE/electrode interface. Copyright 2019, Elsevier. **d** Synthetic scheme for the synthesis of GPR with (1) functionalization of PEO ends, (2) complexation with CD, (3) end capping and simultaneous grafting; ring-opening polymerization of PCL. Copyright 2019, Elsevier

In 2020, Yang et al. [191] systematically investigated the influence of main chains and end groups on the oxidation resistance of PEO using PEG and polyethylene glycol dimethyl ether (PEGDME) with different end groups. The results demonstrated that the *E*_ox_ of –C–O–C– exceeded 4.3 V (vs. Li/Li⁺), whereas –OH was oxidized to –COOH(Li) above 4.05 V (vs. Li/Li⁺), leading to the formation of Li_2_O. This characteristic restricts its compatibility with high-voltage cathodes. The reactive end group –OH is identified as one of the critical factors limiting its application in high-voltage systems (Fig. 17a). By replacing the OH group in PEO with the more stable –OCH₃ group to synthesize PEGDME, and utilizing it as a solid electrolyte in Li||LFP and Li||NCM batteries, capacities of 97% and 90% were retained after 210 and 110 cycles, respectively.

As mentioned in Sect. 3.2.1.1, the substitution of hydrogen atoms with fluorine atoms can enhance the oxidative stability of PEO-based polymers. Recently, Chen et al. [194] conducted a systematic investigation on four types of methoxy poly(ethylene glycol) (MPEG) electrolytes featuring distinct end groups: –OH, propylene carbonate terminal (–PC), methoxy (–OCH_3_), and trifluoroethoxy (–OCH_2_CF_3_). The study revealed that the terminal functional groups significantly influence key physicochemical and electrochemical properties of the MPEG electrolytes, including crystallinity, viscosity, Li salt dissociation efficiency, *t*_+_, ESW, and cycling performance. Among these, MPEG-3F featuring –OCH_2_CF_3_ terminal functional groups demonstrates the highest ionic conductivity. Furthermore, the formation of large-sized aggregates between MPEG-3F and Li salts enables effective encapsulation of TFSI^−^ anions, resulting in the highest *t*_+_. Additionally, the presence of fluorocarbon moieties with high bond energy, endowed with superior high-voltage tolerance, facilitates the expansion of ESW (Fig. 17b). Benefiting from its superior electrochemical performance, this electrolyte with a fluorine-containing functional group significantly enhances the cycling stability of LMBs.

##### Ester Group

Liu et al. [192] prepared a crosslinked polymer solid electrolyte (PPT-SPE) composed of PEO, triethylene glycol dimethacrylate (TEGDMA), and triethylene glycol dimethyl ether (TEGDME) through UV light initiation. Among them, the self-polymerization of TEGDMA forms a rigid linear oligomer (PTEGDMA), which not only enhances the mechanical strength of PPT-SPE but also enhances the intimate contact between the SPE and the electrode by filling the interface voids (Fig. 17c). The presence of methacrylate groups from PTEGDMA and the methoxy groups from TEGDME broadens the ESW to 5.4 V (vs. Li/Li⁺) by reducing the unstable reactivity between –OH terminal group of PEO and electrode. In 2019, Brunklaus et al. [193] reported a hyperbranched graft copolymer (GPR) consisting of a supramolecular structure formed by threading linear PEO into cyclic cyclodextrins (CDs), with simultaneous grafting of polycaprolactone (PCL) via ring-opening polymerization at the PEO terminus and onto the CDs (Fig. 17d). This electrolyte exhibits an ionic conductivity of 1.0 × 10^−3^ S cm^−1^ at 60 °C and exceeds 1.0 × 10^−4^ S cm^−1^ at RT. Furthermore, benefiting from the modification of PCL, this PEO-based electrolyte demonstrated a wide ESW of 4.8 V (vs. Li/Li⁺) and excellent compatibility with NMC (111) cathodes.

#### Others

The incorporation of antioxidant functional groups into PEO-based SPEs, even when these groups are only indirectly associated with the PEO, can lower HOMO energy levels of PEO and significantly enhance the electrolytes' compatibility with high-voltage cathodes [204–207]. Zhan et al. developed a SPE by grafting imidazolium ionic liquids containing –CN groups and PEO onto the backbone of flexible polysiloxane. Benefiting from the low HOMO energy level imparted by –CN group and the capacity of the positively charged imidazolium ionic liquid to increase the amorphous phase fraction of PEO, this cyano-functionalized SPE achieves a high RT ionic conductivity of 3.56 × 10^−4^ S cm^−1^, a high *E*_ox_ of 5.4 V (vs. Li^+^/Li), and excellent cycling performance in Li||LiNi_0.5_Mn_1.5_O_4_ batteries [204]. In 2021, our group developed a cyano-reinforced SPEs by in situ copolymerization of 2-cyanoethyl acrylate and PEG, which enhanced the kinetic oxidation stability of PEO-based SPEs and improved the compatibility with high-voltage LiCoO_2_ via a –C≡N-rich and LiF-rich stable CEI [206]. Recently, Wei et al. [205] designed a grafted SPE using vinylene carbonate as the polymer backbone, with organoboron-modified PEG and acrylonitrile as the grafted segments, thereby extending the oxidation stability window (5 V vs. Li^+^/Li) and facilitating Li^+^ transport (9.24 × 10^−4^ S cm^−1^ at RT).

Through chemical design in molecular level, in summary, utilizing stable functional groups such as methyl, methoxy, cyano, fluorine, or fluorinated groups, as well as some bulky esters to functionalize the EO along the backbone or terminal –OH groups of PEO plays a key role in fundamentally lowering the HOMO energy of electrolytes and constructing robust, LiF-rich CEI, which enhances the interfacial stability toward high-voltage cathodes (over 4.3 V). On this basis, the development of high-voltage-tolerant PEO-based SPEs can truly align with the current demands of the high-energy battery industry, enabling substantial breakthroughs and applications in the power batteries field.

## Conclusion and Outlook

**Fig. 18:**
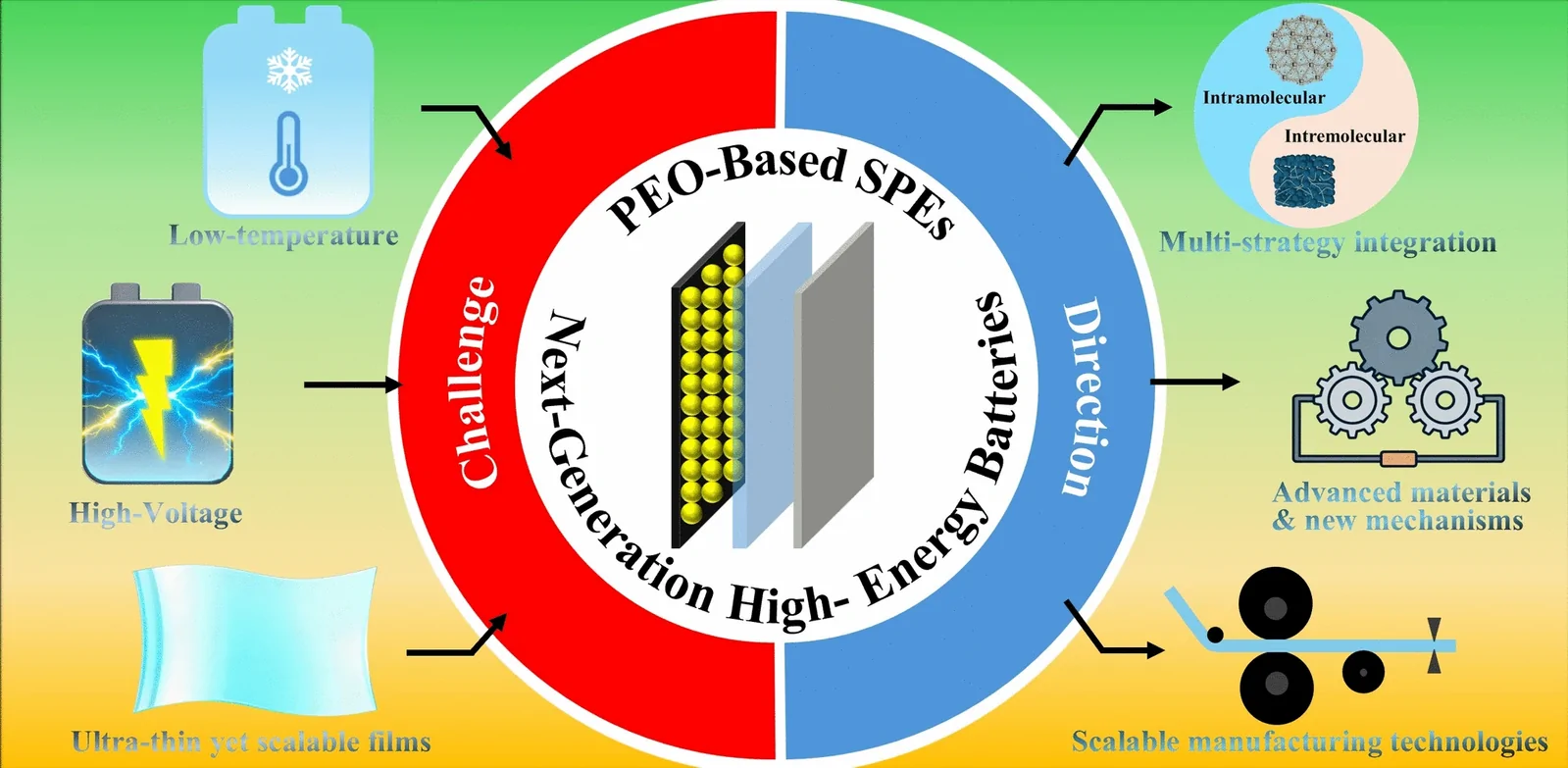
Schematic diagram of current challenges and future research directions for PEO-based SPEs

SPEs are one key component for developing next-generation high-energy and high-safety solid-state batteries. Over the past decade, aiming at commercialization, great efforts were devoted to PEO-based SPEs to tackle their challenges for practical application. While intermolecular strategies, such as GPEs and IPHEs, have been intensely overviewed, this review summarizes the more intrinsic yet fundamental strategy–intramolecular designs, classifying them into topological and chemical methodologies. Encouragingly, current progresses in intramolecular strategy indicate a comprehensive improvement of PEO matrix itself, including not only ionic conductivity and *t*_+_, but also mechanical and high-voltage stabilities, providing a more powerful material for building high-performance SSEs and solid-state batteries. More importantly, through systematic summary, current issues and future research directions of PEO-based SPEs are identified and carefully proposed, respectively (Fig. 18).

1. While introduction of a rigid block can enhance the mechanical strength at elevated temperatures, the linear structure of PEO block and its crystallization feature at RT do not change, leading to a low ionic conductivity at RT (≤ 10^−5^ S cm^−1^). Thus, the application of SPEs based on BCP architecture should focus more on high-temperature scenarios, owing to their better electro-chemo-mechanical stabilities at 60 ~ 150 °C.
2. Crosslinking SPEs exhibit excellent mechanical strength and dendrite resistibility due to their 3D network structure. However, the segmental motion of PEO is also constrained, necessitating plasticizer addition for adequate RT conductivity and electrochemical performance. Using these SPEs to fabricate quasi-solid-state batteries is an important pathway to next-generation high-energy high-safety batteries, despite a failure to achieve all solid state. And it has also demonstrated significant application potential in the field of flexible devices.
3. Brush-like architectures based on short PEO side chains can effectively suppress or eliminate PEO crystallization, increasing ionic conductivity to 10^−4^ S cm^−1^ level at RT. However, such structures intrinsically exhibit low mechanical strength. Combining crosslinking and side-chain grafted strategies to constructing short-brush crosslinked network, which can achieve high mechanical strength while maintain rapid ion transportation, is the most promising SPE route to high-performance all-solid-state LMBs.
4. Applying Li||high-voltage systems to replace current intensely used Li||LFP test model is imperative, which can truly judge the validity of SPEs when serving in high-energy–density LMBs. It has been confirmed that interfacial stability of PEO-based SPEs to high-voltage cathodes can be effectively improved by chemical redesign of PEO molecules, including backbone and terminal modification, with the fundamental mechanisms of either HOMO energy lowering or stable CEI formation. However, these molecule design studies are still relatively limited, which should be more invested.
5. Ideal *t*_+_ of PEO-based SPEs can be achieved by chemically anchoring anionic groups on polymer backbone to fabricate single-ion conductors, which not only mitigates the interfacial side reactions but also suppresses the dendrite growth. In this context, anion centers featuring more delocalized negative charge and ingenious topological modifications are necessary, in order to improve and balance the comprehensive properties required by practical high-energy–density LMBs, such as ionic conductivity, ESW, and even mechanical performance. In fact, although each above intramolecular strategy mainly aims at one specific property, their rational integration to achieve more outstanding battery performance has been implemented and summarized in this review, which will be an important research direction in future.
6. Range anxiety at high-latitude area and winter season is the Achilles’ heel of EV markets, which is attributed to the plummet of ionic kinetics at low temperature. This challenge is further amplified in solid-state batteries. To date, all-solid-state SPEs can hardly touch ionic conductivity beyond 10^−3^ S cm^−1^, translating into a disability in low-temperature operation. Further exploration of advanced materials and new mechanisms should be insistently invested. Merit integration by rationally combining intramolecular and intermolecular strategies is also necessary [210, 211]. Excitingly, very recent report based on a mechanism of “fluorine-oxygen co-coordination” to decouple ionic conductivity to polymer relaxation has achieved 0.27 mS cm^−1^ at − 40 °C, which is relied on the integration of side-chain engineering (topology), fluorochemical modification (chemistry), and solvent additives (intermolecular interaction) [212].
7. Batteries are now increasingly serving in electric vertical take-off and landing (eVTOL) and even human clothing or skins, creating an urgent need for lighter, safer, and more flexible high-energy batteries. However, current thickness of PEO-based SPEs still ranges from 20 to 200 μm, falling far behind commercial liquid electrolyte/separator systems (with the thinnest reaching 3 μm). Considering the large-scale manufacture possibility toward ultra-thin SPE films, with excellent mechanical strength as well as adequate flexibility, is of equal significance to pursuing electrochemical performance in laboratory.

## Abbreviations

ATRP: Atom transfer radical polymerization
BCPs: Block copolymers
BC: Bacterial cellulose
*C*: Cylindrical
CEI: Cathode–electrolyte interphase
CIPs: Contact ion pairs
C1: 3,3′-((Perfluoropropane-2,2-diyl)-bis(4,1-phenylene))bis(3-(trifluoromethyl)-3H-diazirine)
CD: Cyclodextrin
DP: Degree of polymerization
EHPI: 3,6-Epoxy-n-hydroxy-1,2,3,6-tetrahydrophthalimide
EO: Ethylene oxide
*E*_ox_: Oxidation potential
ESW: Electrochemical stability window
EVs: Electric vehicles
FN: Fumaronitrile
FSI^−^: –SO_2_N^(^^−^^)^SO_2__−_
*f*_A_: Volume fraction of block A
*f*_PEO_: Volume fraction of PEO segments
*G*: Double gyroid
*G’*: Storage modulus
GPEs: Gel polymer electrolytes
GPR: Grafted polyrotaxanes
HOMO: Highest occupied molecular orbital
HMDI: 4,4′-Methylenebis(cyclohexyl isocyanate)
*hb*PS: Hyperbranched polystyrene
IPHEs: Inorganic/polymer hybrid solid electrolytes
*L*: Lamellar
LCO: Lithium cobalt oxide
LFP: Lithium iron phosphate
LIBs: Lithium-ion batteries
Li: Lithium
LMBs: Li metal batteries
LiCTFPB: Lithium tetrakis(4-(chloromethyl)-2,3,5,6-tetrafluorophenyl)borate
LiPSTFSI: Lithium poly(4-styrenesulfonyl (trifluoromethanesulfonyl)imide)
LiPSFSI: Lithium poly(styrene sulfonyl fluoride imide)
LiPSsTFSI: Lithium poly(styrene sulfonyl (trifluoromethanesulfonyl)imide)
LiTFSI: Lithium bis(trifluoromethanesulfonyl)imide
LUMO: Lowest unoccupied molecular orbital
LTO: Lithium titanate
mGBCP: Mixed-graft block copolymer
*M*_n_: Molecular weight
MOFs: Metal–organic frameworks
MPEG: Methoxy poly(ethylene glycol)
MPEG-3F: Trifluoroethoxy-terminated methoxy poly(ethylene glycol)
*M*_PEO_: Molecular weight of PEO
NCM: Nickel–cobalt–manganese oxides
PAALi: Lithium polyacrylate
PA: Polyamide
PAN: Polyacrylonitrile
PAM: Polyacrylamide
Pebax: Poly(ether-block-amide)
PC: Polycarbonates
–PC: Propylene carbonate terminal
PCL: Polycaprolactone
PEO: Poly(ethylene oxide)
PEO-Mg-Al-LiTFSI: Mg^2+^- and Al^3+^-coordinated PEO-based electrolyte
PFPM: Poly(3,3,4,4,5,5,6,6,7,7,8,8,9,9,10,10-heptadecafluorodecyl methacrylate)
PFPE: Perfluoropolyethers
PI: Poly(isoprene)
PLA: Poly(lactic acid)
PLiSTFSI: Poly(lithium 4-styrenesulfonyl-(trifluoromethylsulfonyl) imide)
PDMS: Polydimethylsiloxane
PMPCS: Poly(2,5-bis((4-methoxyphenyl)oxycarbonyl)styrene)
PPEGMA: Poly(poly(ethylene glycol) methyl ether methacrylate)
PPMALi: Poly 2-((propionyloxy)methyl) lithium acrylate
PPO: Poly(propylene oxide)
PSt: Polystyrene
PSLi: Lithium poly(styrene sulfonate)
PTMC: Poly(trimethylene carbonate)
TFEMA: Trifluoroethyl methacrylate
PVDF: Poly(vinylidene fluoride)
POSS: Polyhedral oligomeric silsesquioxane
*p*-AGGs: Percolating ionic nanoaggregates
RAFT: Reversible addition-fragmentation chain transfer
ROMP: Ring-opening metathesis polymerization
*r*: Molar ratio of Li⁺ to ethylene oxide unit
RT: Room temperature
*S*: Cubic spherical
SIPEs: Single-ion polymer electrolytes
SIP-in-salt: Single-ion polymer-in-salt electrolyte
SEI: Solid electrolyte interphase
SN: Succinonitrile
SPEs: Solid polymer electrolytes
SSEs: Solid-state electrolytes
*σ*_BCP_: Ionic conductivity of BCP-based SPEs
*σ*_c_: Intrinsic conductivity of the conductive phase (PEO)
TANs: Titanium alcohol networks
TEG: Tetraethylene glycol
TEGDMA: Triethylene glycol dimethacrylate
TEGDME: Triethylene glycol dimethyl ether
*t*_+_: Cationic transference number
*τ*: Sand’s time
*T*_g_: Glass transition temperature
UPy: 2-Ureido-4-pyrimidone
UV: Ultraviolet
ZrMOFs: Zirconium porphyrin-based MOFs
